# Computational analysis of L4–L5 interspinous process devices and interbody fusion spacers using ceramic and polymeric materials via finite element modeling and artificial intelligence

**DOI:** 10.1038/s41598-025-20870-5

**Published:** 2025-10-16

**Authors:** Yomna H. Shash, Rana Hossam Elden

**Affiliations:** Biomedical Engineering Department, Faculty of Engineering, Helwan University, Cairo, Egypt

**Keywords:** Spinal stenosis, Disc degeneration, Interspinous process device, Anterior lumbar interbody fusion, Artificial intelligence, Zirconia, Alumina, PEEK, Engineering, Health care, Materials science, Medical research

## Abstract

Lumbar spinal stenosis involves pathological narrowing of the spinal canal, whereas disc degeneration refers to the progressive deterioration of intervertebral disc structure and function. Interspinous process devices (ISPs) are commonly used to manage spinal stenosis, whereas anterior lumbar interbody fusion (ALIF) spacers are employed for conditions such as spondylolisthesis and disc degeneration. Although titanium remains the standard material for spinal devices, ceramics and polymers have emerged as promising alternatives because of their favorable biomechanical and biological properties. This study investigated the biomechanical performance of ceramic- and polymer-based materials in ALIF and ISP devices via finite element method (FEM) simulations and further explored the potential of artificial intelligence (AI) models to predict biomechanical responses on the basis of material characteristics. In the first phase, FEM models were used to evaluate spinal devices made of ceramics (zirconia, alumina), ceramic composites (ZTA, ATZ), polymers (PEEK, PEKK, PMMA), fiber-reinforced composites (CFR-PEEK, GFR-PEEK), and hydroxyapatite-reinforced PEEK (HA-PEEK) under physiological loading in both normal and osteoporotic bone conditions. In the second phase, eighteen machine learning regression models were trained on FEM-generated datasets to predict key biomechanical metrics, including stress, strain, and range of motion (ROM). The results demonstrated that for ISP devices, stiffer materials such as ceramics and CFR-PEEK 60% reduced stress and strain in adjacent vertebrae compared with softer polymers, thereby lowering fracture risk. Conversely, for ALIF spacers, more compliant materials, such as PEEK and PEKK, offered better performance in osteoporotic spines because of improved load-sharing capabilities. Among AI models, multilayer perceptron (MLP), support vector regression (SVR), and XGBoost achieved high predictive accuracy (R^2^ > 0.95; MAE < 5%), enabling efficient estimation of spinal biomechanics across diverse material and loading scenarios.

**Supplementary Information:**

The online version contains supplementary material available at 10.1038/s41598-025-20870-5.

## Introduction

Lumbar spinal stenosis (LSS), a condition marked by narrowing of the spinal canal or neural foramina leading to nerve compression, is a leading cause of disability and spinal surgeries in individuals over 65 years^1^. It affects around 11% of older adults in the U.S. and over 100 million globally^2,3^. While mild cases respond to conservative therapy, severe stenosis often necessitates surgical decompression, such as laminectomy^4^. Other common degenerative spinal conditions include spondylolisthesis and degenerative disc disease (DDD)^5^. Spondylolisthesis, which is defined by anterior or posterior displacement of a vertebra, has a prevalence of 17.26% in middle-aged adults and increases with age, particularly among women^5–7^. DDD is a progressive condition involving structural degeneration of intervertebral discs and is frequently accompanied by chronic back pain. Radiological signs of DDD are present in 37% of young adults and up to 80% of individuals over the age of 50, with more than 266 million people affected annually^7^. Although conservative management remains the initial approach, surgery is often indicated in refractory or progressive cases^8,9^.

Surgical interventions for these spinal conditions include spinal implants and devices. Interspinous process devices (ISPs) provide minimally invasive treatment for LSS by maintaining spinal motion and indirectly decompressing neural structures^10,11^. Devices like Coflex offer advantages over traditional fusion, including shorter recovery and less invasiveness^12–14^. Anterior lumbar interbody fusion (ALIF) cages, commonly used for degenerative disc disease and spondylolisthesis, restore disc height, promote fusion, and correct spinal alignment through an anterior approach, minimizing disruption to posterior spinal structures^15–17^.

Titanium alloys have traditionally been used in spinal devices due to their superior mechanical strength and long-term durability^13,14,18,19^. However, drawbacks such as MRI imaging artifacts, potential electronic interference, inflammatory or hypersensitivity reactions, and high manufacturing costs have led to growing interest in alternative materials^20–22^. Ceramics, including alumina and zirconia, offer high compressive strength, biocompatibility, corrosion resistance, and radiological compatibility^23–26^. Advanced ceramic composites like zirconia-toughened alumina (ZTA) and alumina-toughened zirconia (ATZ) provide improved fracture toughness and thermal stability, making them viable for spinal applications^27,28^.

Polymers also present attractive alternatives due to their low cost, light weight, ease of manufacturing, and radiolucency^29,30^. Biocompatible polymers such as polyetheretherketone (PEEK), polyetherketoneketone (PEKK), polymethylmethacrylate (PMMA), and ultrahigh-molecular-weight polyethylene (UHMWPE) are widely used in orthopedic and dental implants due to their biocompatibility and shock absorbance ability, though their lower mechanical strength compared to metals and ceramics limits their use in high-load conditions^31–37^. To overcome these limitations, reinforcement strategies have been developed, such as incorporating carbon or glass fibers into polymer matrices (e.g., PEEK) at varying concentrations to yield composites like carbon fiber–reinforced PEEK (CFR-PEEK) and glass fiber–reinforced PEEK (GFR-PEEK) with significantly enhanced strength, stiffness, and wear resistance^38,39^. These improvements help the material better withstand high cyclic loads, resist deformation, and maintain structural integrity over long-term implantation—critical for load-bearing spinal devices subjected to repetitive flexion, extension, and torsional stresses. Additionally, incorporating nanohydroxyapatite (HA) into PEEK (HA-PEEK) improves osteointegration and bone bonding^40–42^, facilitating stronger implant–bone interfaces and potentially reducing the risk of loosening or migration. Reinforcement contents of 30% and 60% are frequently reported in the literature for PEEK-based composites, as they offer substantial improvements in stiffness and strength while maintaining acceptable processing characteristics and biocompatibility^38–42^.

Despite substantial progress in spinal biomechanics research, most studies have primarily focused on conventional materials such as titanium alloys, typically evaluating a single device type—most often anterior lumbar interbody fusion (ALIF) cages—or examining a limited set of loading conditions. Consequently, there is a lack of comprehensive, comparative biomechanical evaluations that examine emerging materials—including ceramics, fiber-reinforced composites, and advanced polymers—across multiple spinal device configurations and in diverse bone quality scenarios, such as osteoporosis. Furthermore, no prior work, to our knowledge, has simultaneously assessed both anterior and posterior spinal devices under multiple physiological motions while incorporating AI predictive modeling tools.

To address these gaps, this study introduces a two-phase computational framework that integrates finite element analysis (FEA) with artificial intelligence (AI) regression modeling. In the first phase, detailed finite element analyses (FEA) were conducted on two distinct spinal devices—anteriorly placed anterior lumbar interbody fusion (ALIF) cages and posteriorly placed interspinous process (ISP) devices—made from a broad range of advanced materials. These included ceramics (zirconia, alumina, zirconia-toughened alumina [ZTA], alumina-toughened zirconia [ATZ]), polymers (polyetherketoneketone [PEKK], polyetheretherketone [PEEK], polymethylmethacrylate [PMMA]), and PEEK-based composites with varying reinforcement types and contents (carbon fiber–reinforced PEEK [CFR-PEEK] at 30% and 60% fiber content, glass fiber–reinforced PEEK [GFR-PEEK] at 30% and 60% fiber content, and hydroxyapatite-reinforced PEEK [HA-PEEK] at 30% and 60% filler content). Simulations were performed under both normal and osteoporotic bone conditions across multiple physiological motions. Key biomechanical outputs included implant stress, stress and strain in adjacent vertebrae, and segmental range of motion.

**Fig. 1:**
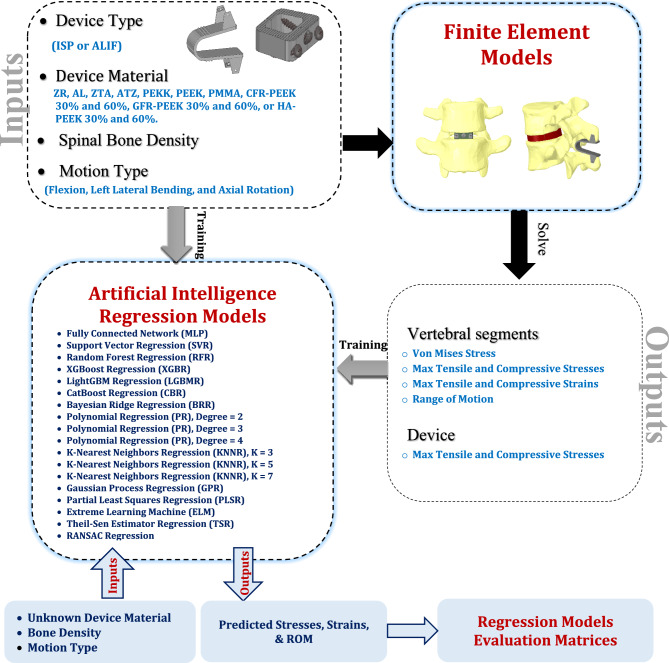
Workflow depicting the integration of finite element analysis with AI models. ISP: interspinous process device, and ALIF: anterior lumbar interbody fusion.

In the second phase, AI-based regression models—encompassing linear, nonlinear, ensemble, kernel-based, and neural network approaches—are developed using the FEA-generated datasets to rapidly predict biomechanical outcomes based on material properties, loading conditions, and bone quality. This combined FEA–AI methodology enables fast, accurate prediction of device performance without re-running full simulations, offering a scalable and patient-specific tool for material selection^43–48^. By systematically comparing a broad spectrum of advanced materials across two device types and integrating AI-driven predictive modeling, this work represents a novel, clinically translatable approach that bridges the gap between computational biomechanics and personalized spinal device design. The overall methodological framework is illustrated in Fig. 1.

The null hypothesis of this study is that certain advanced ceramics, fiber-reinforced composites, and high-performance polymers demonstrate biomechanical performance comparable to or exceeding that of titanium alloys in both anterior lumbar interbody fusion (ALIF) cages and interspinous process (ISP) devices, and that AI-based regression models can accurately predict key mechanical outcomes from material properties, bone quality, and loading conditions. Validating this hypothesis will support the accelerated development of next-generation spinal devices and enable evidence-based, patient-specific material selection to improve clinical outcomes.

## Materials and methods

### Geometry preparation and finite element model construction

#### 3D modeling of spinal devices

Three finite element models of the lumbar spine (L1–L5) were constructed via SpaceClaim (a 3D modeling software by Ansys) and analyzed in ANSYS 18 (Ansys Inc., Canonsburg, USA) to simulate healthy and pathological spinal conditions. These included an intact lumbar spine, a spine implanted with an interspinous process device (ISP), and one with an anterior lumbar interbody fusion (ALIF) spacer, representing common surgical treatments for lumbar spinal stenosis and disc degeneration^11–17^. Simulations were performed under both normal and osteoporotic bone density conditions to evaluate how bone quality influences spinal biomechanics and device performance.

**Fig. 2:**
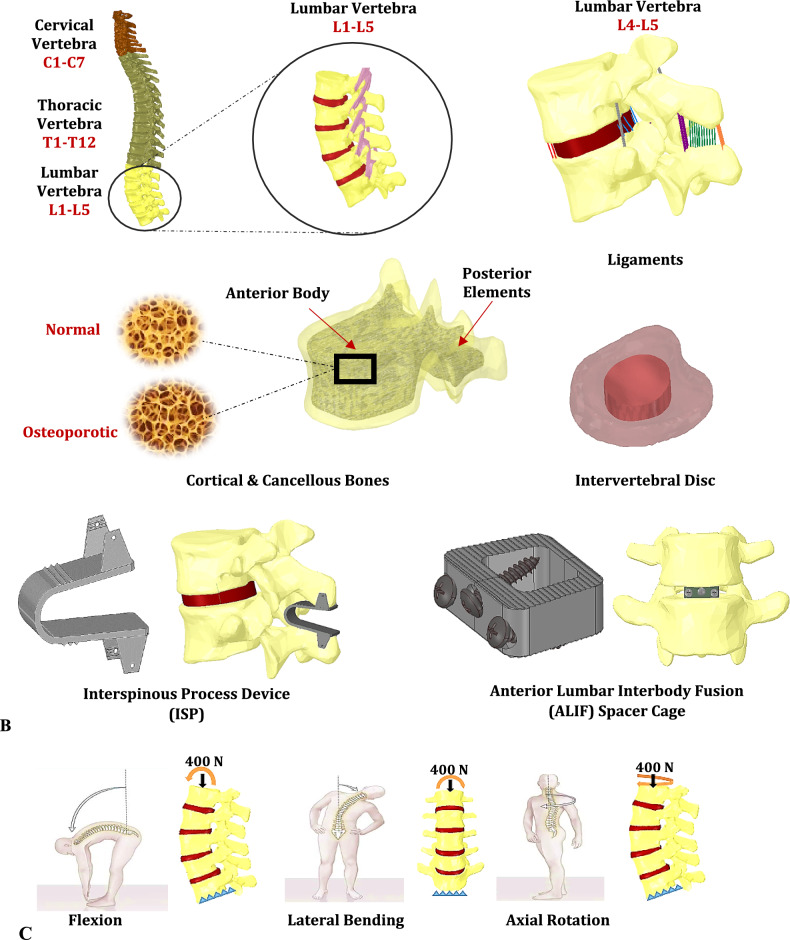
(**A**) Complete model of the intact lumbar spine. (**B**) Lumbar treatment via the ISP device and stand-alone ALIF cage and (**C**) Loading conditions and constraints on the lumbar L1-L5 segment.

The anatomical geometry of the lumbar spine was obtained in OBJ format from an open-access online dataset (BodyParts3D, Life Sciences Integrated Database Center, Japan), comprising vertebrae, intervertebral discs, and major supporting structures^49^. Using SpaceClaim software (ANSYS® SpaceClaim, Version 18.0, ANSYS Inc., Canonsburg, PA, USA^50^), each vertebra was reconstructed with a 2 mm-thick cortical shell surrounding the cancellous bone, as shown in Fig. 2A ^51^. The intervertebral discs were modeled with anatomically distinct regions representing the nucleus pulposus and annulus fibrosus^13,52^. Additionally, 1 mm-thick cartilaginous endplates were incorporated at the vertebra-disc interfaces and coupled through tie contact conditions to ensure anatomical continuity and load transfer^52^. Facet joints were modeled with frictional contact (coefficient = 0.1) to simulate realistic load transfer, joint stability, and articulation^52^. Seven primary spinal ligaments—including the anterior and posterior longitudinal ligaments; the ligamentum flavum; and the intertransverse, capsular, interspinous, and supraspinous ligaments—were used to mimic physiological spine behavior^37,52^.

Two spinal devices targeting the L4–L5 segment were developed and integrated into the finite element model. The interspinous process (ISP) device (Fig. 2B), designed to alleviate lumbar spinal stenosis while preserving segmental motion, measured 25 mm in length and 10 mm in width. It featured 1 mm serrated teeth to enhance implant stabilization and reduce the risk of loosening or bone damage over time. The ISP device was positioned between adjacent spinous processes, with surface-to-surface contact and a frictional interface (coefficient = 0.8) applied at the device-bone interface to simulate mechanical interlock and mitigate implant migration^11–14^.

The anterior lumbar interbody fusion (ALIF) spacer, intended to restore disc height and address degenerative disc disease or low-grade spondylolisthesis, measured 30 mm in length and 8 mm in height. It incorporated stepped anchoring teeth for fixation to adjacent endplates, a central aperture for packing autologous cancellous bone graft, and three lateral holes designed to accommodate 3 mm-diameter fixation screws, promoting intersegmental stability. In the ALIF models, screw-to-bone interfaces were defined using tie constraints, representing fully bonded conditions with no relative motion, thereby isolating the mechanical influence of the cage material. The contact between the ALIF cage and vertebral endplates was modeled using surface-to-surface contact with a friction coefficient of 0.2, allowing for controlled load transfer without penetration^15–17^.

#### Loading and boundary conditions

A static structural analysis was conducted using ANSYS to evaluate the biomechanical response of the lumbar spine and implanted devices under physiological conditions. A compressive preload of 400 N was applied vertically to the superior endplate of the L1 vertebra to simulate upper body weight transmission. Additionally, a 200 N follower load was implemented along the curvature of the lumbar spine to replicate the stabilizing effect of paraspinal muscle forces while maintaining spinal alignment under compression^13^.

To simulate daily functional movements, pure moments of 8 Nm were applied at the superior surface of L1 in three principal directions—flexion, lateral bending, and axial rotation—representing standardized biomechanical testing protocols for spinal segments^13,52^. The inferior surface of the L5 vertebra was rigidly fixed in all degrees of freedom (translations and rotations in X, Y, and Z axes) to provide a stable boundary condition, as depicted in Fig. 2C. All loading conditions were applied incrementally in a quasi-static manner to avoid inertial effects and ensure convergence.

#### Mesh setting

A high-resolution finite element mesh was generated in ANSYS using an adaptive sizing function to accurately capture geometric complexity and stress gradients. All components from L1 to L5 were discretized using three-dimensional tetrahedral elements. Ligaments were modeled independently as tension-only truss elements and were excluded from the mesh density assessment due to their simplified representation.

**Fig. 3:**
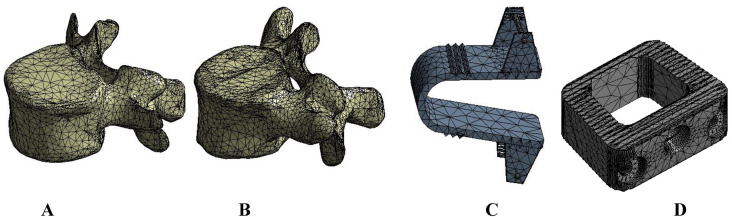
Meshing of: (**A**) L4 vertebra, (**B**) L5 vertebra, (**C**) ISP device and (**D**) ALIF cage.

To ensure numerical reliability, a mesh convergence study was conducted by evaluating the maximum von Mises stress across varying mesh densities. The convergence behavior was assessed by plotting stress values against the total number of elements for key model configurations, including the ISP device, ALIF cage, fixation screws, and the intact L4–L5 disc and vertebrae under flexion loading (see Figure S1). Mesh convergence was deemed achieved when changes in maximum von Mises stress remained within 5% across successive refinements, ensuring a balance between computational efficiency and solution accuracy. Element and node counts for selected anatomical and device components are summarized in Table 1. Figure 3 illustrates representative meshes for the L4 and L5 vertebrae, the ISP device, and the ALIF cage. To maintain consistency and enable direct comparison across simulation scenarios, the finalized mesh settings were uniformly applied to all loading conditions.

**Table 1:** The number of elements and nodes in meshing.

	No. of elements	No. of nodes
L4 vertebra	32,636	57,251
L5 vertebra	35,064	61,183
ISP device	7212	12,766
ALIF cage	26,704	46,932
Fixation screws	83,432	112,984
L4-5 intervertebral disc	4680	7561

#### Material properties

The material properties of both healthy and osteoporotic spines were obtained from the literature and are summarized in Table 2. To represent osteoporotic conditions, the elastic modulus of both cancellous and cortical bone tissues was reduced according to values reported in previous studies^13,14,53–58^. Microstructural alterations such as trabecular thinning, loss of connectivity, and increased porosity were not explicitly modeled. This simplification follows commonly adopted finite element modeling practices for simulating osteoporotic bone but may not fully capture local variations in trabecular morphology or heterogeneous load transfer at the bone–implant interface.

**Table 2:** The properties of normal and osteoporotic vertebrae.

	Young’s modulus (MPa)			Poisson’s ratio	References
	X	Y	Z		
Normal model					
Cortical bone	12,000	12,000	22,000	0.30	^53–58^
Cancellous bone	100	100	450	0.20	
Posterior element	3500	3500	3500	0.25	
Osteoporotic model					
Cortical bone	8040	8040	12,000	0.30	^53–58^
Cancellous bone	34	34	300	0.20	
Posterior element	2345	2345	2345	0.25	

Under osteoporotic conditions, cortical bone strength was reduced from approximately 140 MPa (tensile) and 200 MPa (compressive) to 90 MPa and 120 MPa, respectively, whereas cancellous bone strength decreased from roughly 10–12 MPa to 3–3.5 MPa^56–58^. Both cortical and cancellous bone tissues were modeled as orthotropic materials to represent their anisotropic mechanical behavior and directional stiffness^53^.

The nucleus pulposus was simulated via a Mooney‒Rivlin hyperelastic incompressible fluid model^13^, whereas the annulus fibrosus was treated as a hyperelastic solid (Table 3)^13,54^. Seven spinal ligaments—the anterior longitudinal ligament (ALL), posterior longitudinal ligament (PLL), ligamentum flavum (FL), capsular ligament (CL), interspinous ligament (ISL), supraspinous ligament (SSL), and transverse ligament (TL)—were simplified as linear springs with stiffness values of 7.8 MPa, 10 MPa, 15 MPa, 7.5 MPa, 10 MPa, 8 MPa, and 10 MPa, respectively, and poison ratios of 0.4^54^.

**Table 3:** The properties of intervertebral discs obtained via the Mooney‒Rivlin formulation.

	Young’s modulus (MPa)		Poisson’s ratio	References
Endplate	25		0.25	
Nucleus pulposus	*C01* = 0.12	*C10* = 0.03	–	^13^
Annulus substance	*C01* = 0.18	*C10* = 0.045	–	
Annulus fibers	550		0.30	

**Fig. 4:**
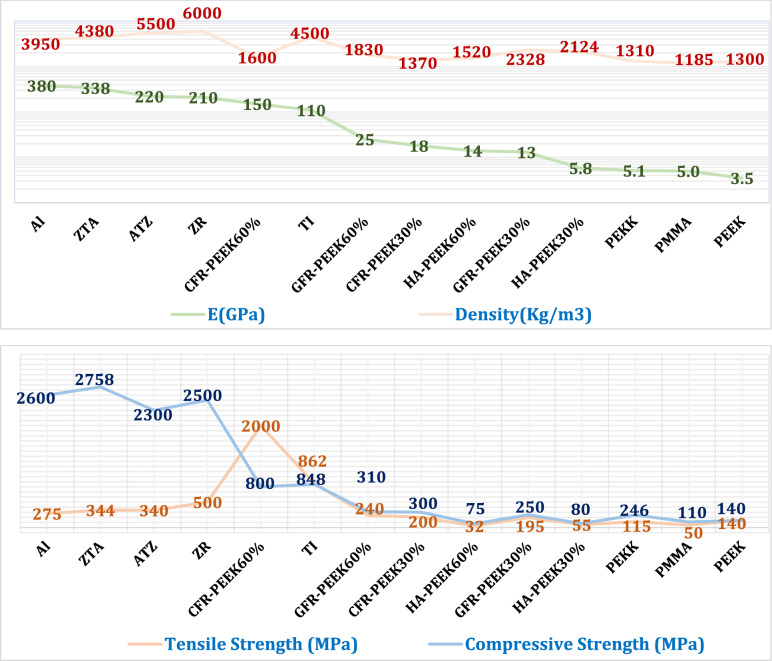
Elastic modulus (GPa), density (kg/m^3^), and tensile and compressive strengths (MPa) of different materials^27,28,35,37,38,40,41,59–64^.

Titanium served as the baseline material for both the interspinous process (ISP) devices and anterior lumbar interbody fusion (ALIF) cages, with all fixation screws similarly modeled using titanium. To evaluate alternative biomaterials, the simulations incorporated a wide range of candidates, including ceramics (zirconia, alumina), ceramic composites (zirconia-toughened alumina [ZTA], alumina-toughened zirconia [ATZ]), polymers (PEEK, PEKK, PMMA), fiber-reinforced composites (carbon- and glass-fiber-reinforced PEEK [CFR-PEEK, GFR-PEEK] with 30% and 60% fiber content), and hydroxyapatite-reinforced PEEK (HA-PEEK with 30% and 60% HA content). The corresponding mechanical properties—such as elastic modulus, density, and ultimate strength—were extracted from peer-reviewed literature and are summarized in Fig. 4^27,28,35,37,38,40,41,59–64^.

For ISP and ALIF devices, all materials were modeled as linear elastic and isotropic to maintain computational stability, ensure comparability across material types, and isolate the influence of elastic modulus and Poisson’s ratio on biomechanical response—avoiding variability from complex constitutive behavior. This approach aligns with established practices in spinal finite element modeling^13,14,17,18,29,37,52–54^. While certain materials (e.g., ceramics, fiber composites) exhibit brittle or anisotropic behavior in reality, the present study focused on comparative performance under quasi-static conditions. Consequently, fracture mechanics, anisotropy, and time-dependent effects (e.g., creep, fatigue) were not considered. Instead, stress distributions were qualitatively compared with reported yield strengths to infer potential failure risks and inform material selection for spinal device design.

#### Biomechanical outputs

Key biomechanical outputs—including principal stresses, principal strains, and range of motion (ROM)—were extracted from the finite element simulations to evaluate implant performance and bone response. Device integrity was assessed by comparing the maximum (tensile) and minimum (compressive) principal stresses against the respective material yield strengths in tension and compression (as summarized in Fig. 4), to identify potential regions of failure or material overstress^53,57,65,66^.

Vertebral bone tissues were analyzed using the principal stress failure criterion, reflecting their distinct mechanical characteristics: brittle failure behavior in cortical bone and more ductile behavior in cancellous bone^57,65^. Principal stresses in each region were compared to corresponding tensile and compressive strength values to assess the risk of structural compromise.

In addition to stress-based assessments, principal strain values in cortical and cancellous bone were evaluated to infer bone remodeling potential. Based on mechano-biological thresholds, cortical bone strains below 200 microstrain (με) may indicate a risk of disuse-induced resorption, while strains exceeding 3,000 με could suggest overloading, remodeling imbalance, or the potential for fatigue failure under repetitive loading, consistent with Wolff’s law^67^. Cancellous bone, due to its greater deformability, exhibits a wider physiological strain tolerance, with acceptable strain levels typically ranging between 500 and 7,000 με^68^.

### Machine learning-based prediction of spinal biomechanical responses

A suite of regression-based machine learning models was developed in MATLAB R2023b (MathWorks Inc., Natick, MA, USA) to predict the biomechanical responses of spinal implants—specifically, stress, strain, and range of motion (ROM)—as functions of material properties, vertebral bone quality, and applied loading conditions derived from finite element (FEM) simulations. Two distinct datasets were constructed, corresponding to the interspinous process (ISP) device and the anterior lumbar interbody fusion (ALIF) cage. Each dataset incorporated a wide range of biomaterial properties, six standardized loading conditions—flexion, extension, left and right lateral bending, and left and right axial rotation—and two bone quality categories: normal-density and osteoporotic vertebrae.

#### Regression models

The modeling framework incorporated a diverse set of machine learning algorithms, including classical statistical methods, kernel-based approaches, ensemble learning techniques, neural networks, and probabilistic models—each selected to effectively address the nonlinear, high-dimensional nature of biomechanical data. Among these, the multilayer perceptron (MLP) was employed for its capacity to capture complex nonlinear relationships through deep, multilayer architectures and nonlinear activation functions^69^. Support vector regression (SVR) with a radial basis function (RBF) kernel was utilized for its strong generalization ability in high-dimensional feature spaces and its suitability for modeling nonlinear, anisotropic mechanical behaviors^70^. Random forest regression was included for its robustness against overfitting and noise, leveraging ensemble averaging and decision tree diversity to enhance predictive stability^71,72^.

Gradient boosting methods—including XGBoost, LightGBM, and CatBoost—were incorporated for their computational efficiency, advanced regularization, and improved handling of categorical interactions^73–75^. Bayesian ridge regression offers a principled framework to manage multicollinearity and quantify uncertainty in parameter estimation, enhancing model interpretability^76^. Polynomial regression was applied at varying degrees to capture nonlinear stress‒strain relationships in a straightforward manner^77^. K-nearest neighbor regression (KNNR) offers a nonparametric approach capable of capturing localized data patterns without assuming a global model structure^78^. Gaussian process regression (GPR) provides both predictions and uncertainty quantification, which is critical for safety and risk assessments in biomedical applications^79^. Partial least squares regression (PLSR) addresses high dimensionality and multicollinearity by extracting latent variables that maximally covary with the biomechanical output metrics^80^.

The extreme learning machine (ELM) enables fast training and strong generalization by leveraging randomly initialized single-layer networks, making it well suited for large FEM-derived datasets^81^. To increase robustness against noise and outliers, the Theil–Sen estimator was employed for consistency in the presence of nonGaussian error distributions^82^, and RANSAC regression was used to identify the most consistent data subsets for reliable model fitting in noisy simulation environments^83^. Collectively, this diverse modeling framework provides a robust, interpretable, and computationally scalable solution for accurately predicting spinal device biomechanics across a wide range of anatomical conditions, material types, and loading scenarios.

#### Evaluation matrices

To comprehensively evaluate the performance and reliability of the regression models developed for predicting the biomechanical responses of ALIF and ISP spinal devices, five widely recognized evaluation metrics were employed: the coefficient of determination (R^2^), mean absolute error (MAE), mean squared error (MSE), root mean squared error (RMSE), and computational efficiency. The R^2^ metric quantified the proportion of variance in the FEM-derived outputs that was captured by the model predictions, serving as a primary indicator of predictive accuracy and explanatory power^84^.

1 $${R}^{2}=1-\frac{\sum_{i=1}^{n}{\left({y}_{i}-\widehat{{y}_{i}}\right)}^{2}}{\sum_{i=1}^{n}{\left({y}_{i}-\widehat{{y}_{i}}\right)}^{2}}$$

The MAE measures the average magnitude of absolute prediction errors, offering an intuitive and interpretable assessment of overall model accuracy while maintaining robustness to outliers^85^. In contrast, the MSE emphasized larger errors by squaring the deviations, thereby penalizing extreme discrepancies more heavily. This sensitivity to large errors is particularly important in biomechanical applications, where substantial deviations in stress or strain predictions may have critical clinical implications.

2 $$MAE=\frac{1}{n}\sum_{i=1}^{n}\left|{y}_{i}-\widehat{{y}_{i}}\right|$$

The RMSE, defined as the square root of the MSE, expresses prediction errors in the same units as the target biomechanical metrics (e.g., stress, strain, or displacement), thereby enhancing interpretability and clinical relevance^86^. These error-based metrics—MAE, MSE, and RMSE—enabled quantitative assessment of how closely each model’s predictions approximated the ground truth FEM outputs across varying anatomical levels, material types, and loading conditions. In addition, computational efficiency was evaluated to determine the feasibility of deploying each model in real-time or near-real-time simulation environments, a critical consideration for clinical and design applications. Collectively, these evaluation criteria provide a comprehensive framework for assessing the predictive accuracy, robustness, and practical applicability of the machine learning models employed in spinal device performance prediction.

3 $$RMSE=\sqrt{\frac{1}{n}\sum_{i=1}^{n}{\left({y}_{i}-\widehat{{y}_{i}}\right)}^{2}}$$

The execution time refers to the time required by each regression model to perform training and generate predictions. Although this metric does not measure prediction accuracy directly, it is critical when considering computational efficiency—especially for large-scale simulations or real-time surgical planning applications. In this study, the execution time is recorded and compared across all the models to identify those best suited for fast, scalable deployment in clinical or engineering settings.

The dataset was partitioned using Hold-Out strategy into 80% for training and 20% for independent testing, with strict separation to prevent test data from influencing model development or hyperparameter optimization. Model performance was reported exclusively on the independent test set to ensure unbiased evaluation, confirm generalization capability, and mitigate the risk of overfitting.

## Results

### Finite element model

#### Validation study

**Fig. 5:**
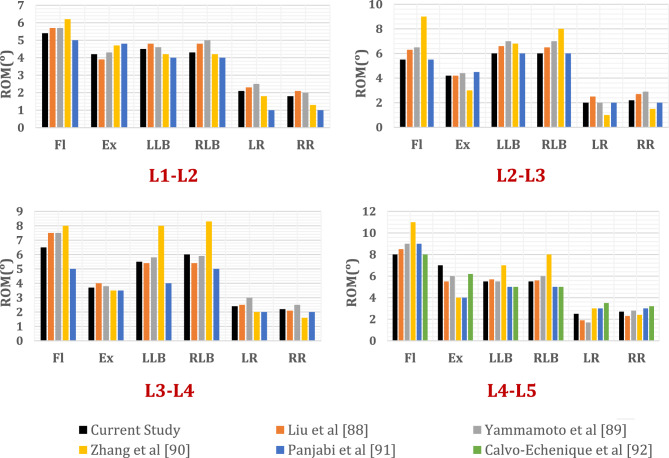
Comparison of the ROM of each motion segment of L1-L5 between the current and previous experimental and computational studies, under 10 N m moment.

The intact lumbar spine finite element model was validated against previously published experimental cadaveric data and established computational biomechanical studies to confirm its anatomical fidelity and predictive accuracy^87–91^. For validation of the normal-density spine (Fig. 5), a pure moment of 10 Nm was applied to the superior endplate of the L1 vertebra in six standard loading directions commonly used in spinal biomechanics: flexion (FL), extension (EX), left lateral bending (LLB), right lateral bending (RLB), left axial rotation (LR), and right axial rotation (RR). The inferior surface of the L5 vertebra was fully constrained in all six degrees of freedom (three translations and three rotations) to simulate a fixed base. The resulting segmental ranges of motion (ROMs) were extracted and compared with published experimental data. As shown in Fig. 5, the ROM values closely matched those reported in the literature, with minor variations attributable to individual anatomical differences and simplifications inherent in the finite element modeling process.

**Fig. 6:**
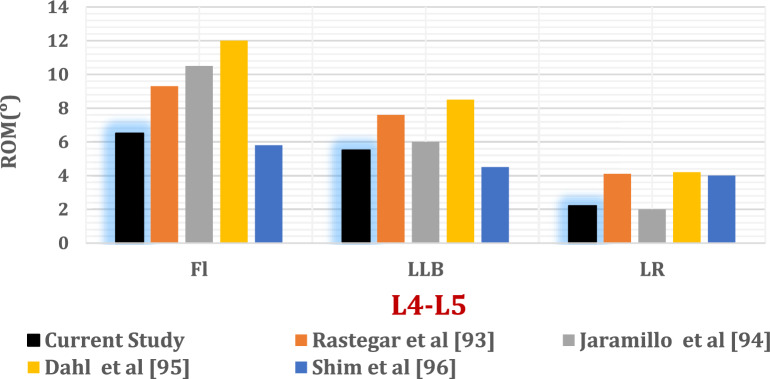
Comparison of the ROM of L4-L5 segment between the current and previous experimental and computational studies, under 8 N m moment.

In addition to whole-spine validation, the L4–L5 functional spinal unit (FSU) was independently assessed by applying a reduced pure moment of 8 Nm in flexion, lateral bending, and axial rotation. The corresponding ROM values (Fig. 6) showed strong agreement with those obtained in prior cadaveric experiments and validated finite element studies^92–95^, further supporting the model’s biomechanical validity at the segmental level.

This multi-level validation approach strengthens the credibility of subsequent implant simulations by ensuring that the model realistically replicates physiological spinal motion under standardized loading conditions.

#### Sensitivity analysis

A local sensitivity analysis was performed on the intact lumber model to evaluate the robustness of model predictions. Key parameters, including moment magnitude (± 10%), facet joint friction (0.1–0.3), disc stiffness (± 20%), and L5 constraint type (full vs. partial fixation), were individually varied while monitoring changes in L4–L5 ROM and maximum von Mises stress (MPa). All other model parameters remained constant during each perturbation.

Sensitivity analysis results are summarized in Table S1. Among tested parameters, changes in bending moment had the highest influence on both stress (± 6.1%) and ROM (± 6.9%), as expected. Variations in facet joint friction and disc stiffness induced moderate changes in stress and ROM (within ± 5%), while the choice of L5 constraint conditions resulted in minor effects (< 3%). These findings suggest that the model predictions were stable across a range of physiologically relevant input variations.

#### Comparisons between intact spines and diseased spines rehabilitated with ISP device or stand-alone ALIF cage

**Fig. 7:**
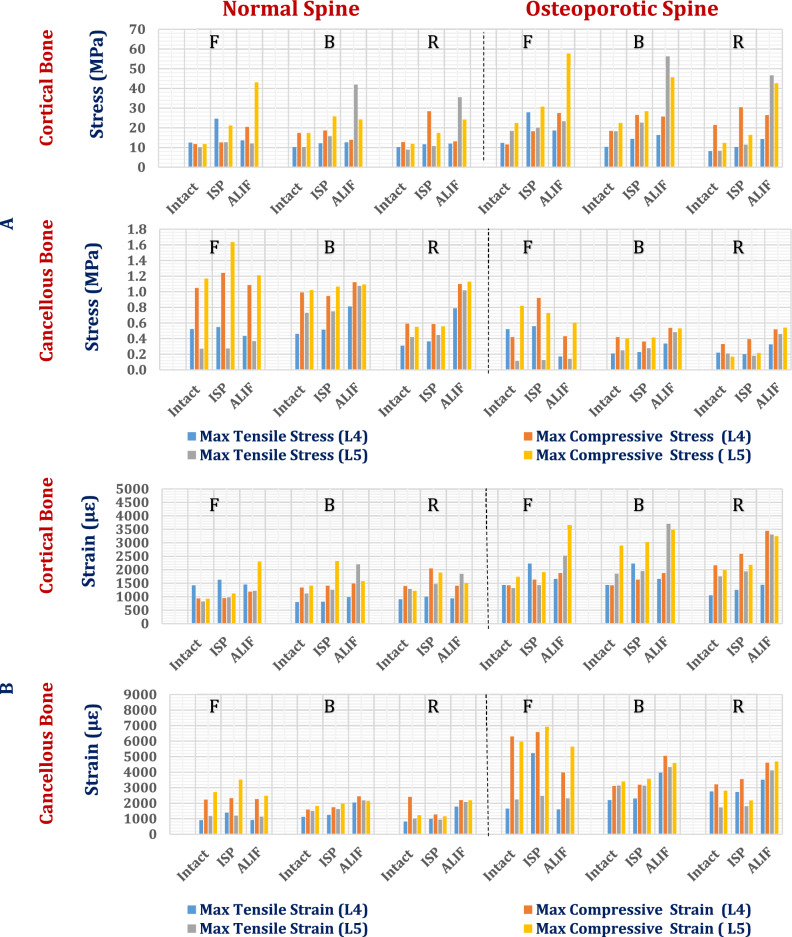
Maximum tensile and compressive stresses (**A**) and strains (**B**) of cortical and cancellous bones in the intact model and pathological models rehabilitated with ISP and standalone ALIF in flexion (F), bending (B), and rotation (R).

Figure 7 shows the maximum tensile and compressive stresses and strains in the cortical and cancellous bone of the L4 and L5 vertebrae across intact and surgically treated lumbar spine models. These models were evaluated under flexion, lateral bending, and axial rotation following implantation of titanium ISP or ALIF devices, considering both normal and osteoporotic bone conditions.

Both ISP and ALIF treatments induced increased cortical bone stresses and strains relative to those in the intact spine. Notably, in the L5 vertebra, the ALIF configuration generated compressive strains exceeding 2300 με in the normal bone model and 3500 με in the osteoporotic model. Cancellous bone strains remain within physiological limits, generally below 4000 με in normal models and 7000 με in osteoporotic models.

As shown in Fig. 7, the intact lumbar spine—with all native structures preserved, including intervertebral discs, ligaments, and facet joints—demonstrated more efficient load distribution, resulting in lower and more uniform vertebral stresses. In contrast, the standalone ALIF model presented the highest cortical bone stress levels, particularly at L5, due to the absence of posterior elements and increased reliance on anterior column support. Notably, high stresses were observed at the cage–endplate interface and around the screw insertion sites, contributing to elevated stress values in the ALIF-treated models compared with those in the ISP and intact configurations.

#### Stenosis treatment with interspinous process devices *(ISPs)*

##### Stresses on ISPs

**Fig. 8:**
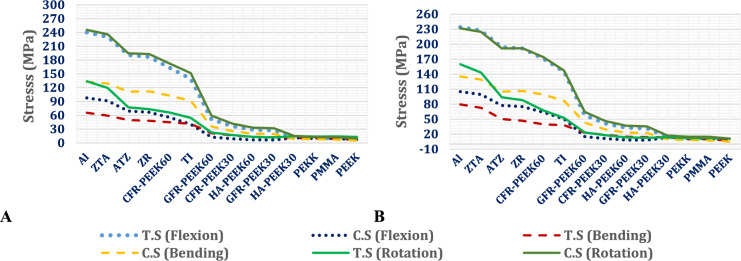
Maximum tensile (T.s.) and compressive (C. S) Stress on ISPs with various materials: (**A**) Normal spine and (**B**) Osteoporotic spine.

**Fig. 9:**
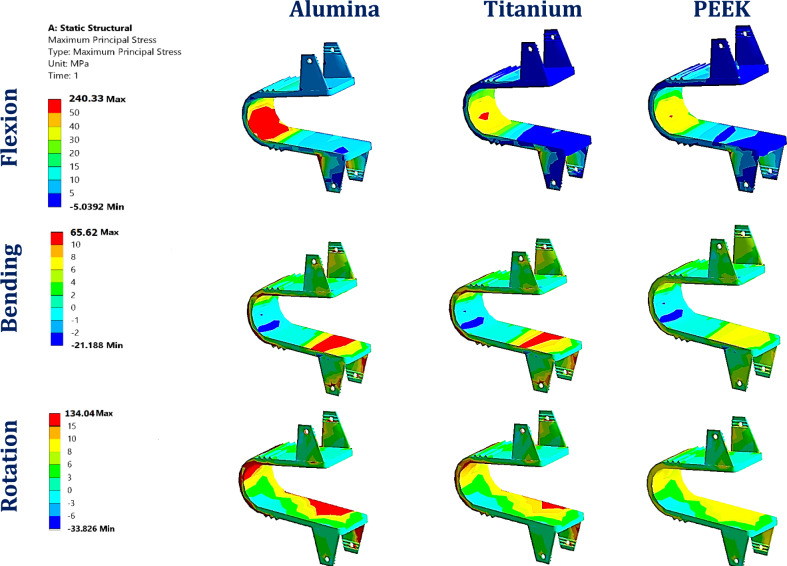
Distribution of maximum principal stresses (MPa) on alumina, titanium, and PEEK ISPs in normal spines.

Figure 8 depicts the maximum tensile and compressive stresses in interspinous process devices (ISPs) constructed from a various material, ranging from high-stiffness ceramics (e.g., alumina) to low-stiffness polymers (e.g., PEEK), assessed within both normal and osteoporotic lumbar spine models. Figure 9 shows the distributions of the maximum principal stresses in the alumina, titanium, and PEEK ISPs under flexion, lateral bending, and axial rotation loading in the normal spine model.

In the normal spine model, titanium ISPs exhibited maximum tensile and compressive stresses of 139 MPa and 40.6 MPa during flexion, 41.38 MPa and 91.5 MPa during lateral bending, and 54.5 MPa and 151.94 MPa during axial rotation, respectively. Substitution with ceramic ISPs led to notable stress increases, exceeding 34% (tensile) and 63% (compressive) during flexion, 16.5% and 22% during lateral bending, and 34% and 27% during axial rotation. In the osteoporotic model, titanium ISPs presented tensile and compressive stresses of 144.88 MPa and 50.74 MPa in flexion, 37.82 MPa and 86.8 MPa in bending, and 52.6 MPa and 147.13 MPa in rotation. The corresponding ceramic ISP-induced stress increases exceed 32% (tensile) and 48% (compressive) during flexion, 23.8% and 22.5% during bending, and 67% and 30% during rotation, respectively.

The CFR-PEEK ISP with 60% fiber content demonstrated moderate stress elevations, remaining below 36% in the normal spine and 28% in the osteoporotic spine. In contrast, PEEK-based composites—including CFR-PEEK (30%), GFR-PEEK (30% and 60%), and HA-PEEK (30% and 60%)—substantially reduced tensile and compressive stresses under all loading conditions, achieving reductions exceeding 60% in flexion, up to 60% in lateral bending, and more than 55% in axial rotation for both spine conditions.

Notably, ISPs manufactured from softer polymers such as PEKK, PMMA, and PEEK exhibited the greatest stress mitigation, reducing the maximum tensile and compressive stresses by more than 90% and 75%, respectively, relative to those of titanium devices across all loading modes. These effects were consistent in both normal and osteoporotic models.

Endurance analysis confirmed that all ISP materials remained within safe biomechanical limits under physiological loads. However, the HA-PEEK device with 60% reinforcement approached its tensile yield strength (32 MPa) during flexion in the osteoporotic spine, suggesting a localized risk of mechanical failure under high-strain conditions.

##### Stresses and strains on the L4 and L5 segments via the ISPs

**Fig. 10:**
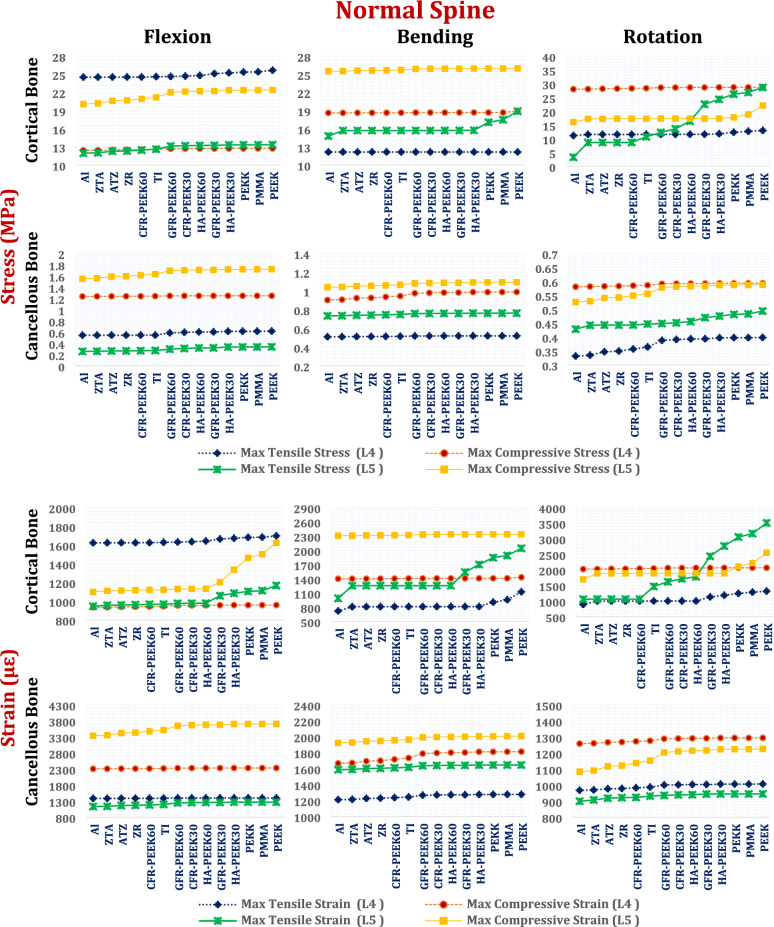
Tensile and compressive stress (MPa) and strains (µԑ) on the cortical and cancellous bones of the L4 and L5 segments via ISPs in normal spines.

**Fig. 11:**
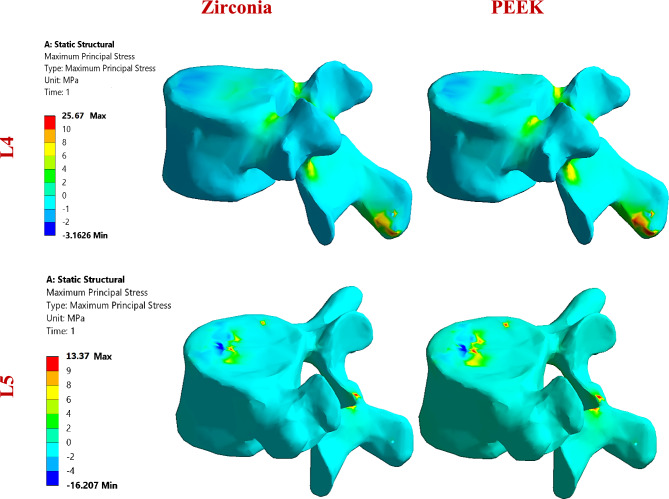
Distribution of maximum principal stresses (MPa) on the cortical bones of the L4 and L5 segments via zirconia and PEEK ISPs in normal spines, under flexion.

**Fig. 12:**
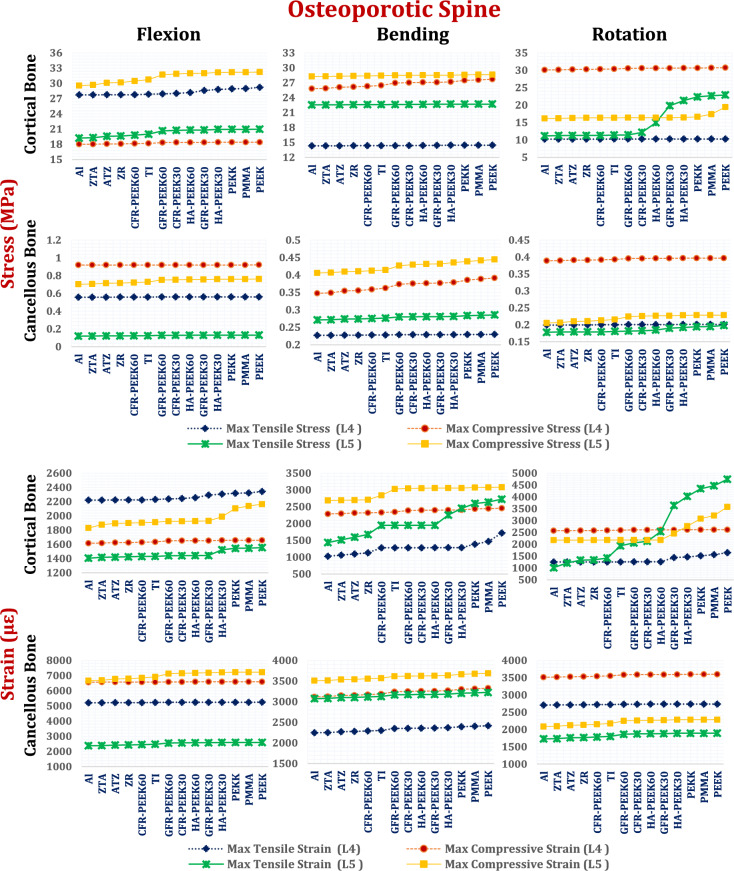
Tensile and compressive stress (MPa) and strains (µԑ) on the cortical and cancellous bones of the L4 and L5 segments via ISPs in osteoporotic spines.

Maximum tensile and compressive stresses and strains in the cortical and cancellous bones of the L4 and L5 vertebrae were analyzed in both normal and osteoporotic lumbar spine models via interspinous process devices (ISPs) composed of ceramic and polymeric materials as alternatives to titanium, as shown in Figs. 10, 11 and 12.

In the normal spine under flexion (Fig. 10), titanium ISPs produced cortical bone stresses of up to 24.6 MPa at L4 and 12.63 MPa at L5, with corresponding tensile strains of 1629 µε and 1122 µε, respectively. The ceramic and CFR-PEEK 60% devices slightly reduced these values (< 5.5%), whereas the CFR-PEEK 30%, GFR-PEEK, and HA-PEEK devices caused minor increases (< 6%). In contrast, soft polymeric ISPs such as PEEK, PEKK, and PMMA led to the highest stress and strain levels. During lateral bending, titanium devices generated cortical stresses of 18.65 MPa at L4 and 25.73 MPa at L5. Ceramic materials reduced these values by up to 21.3%, whereas CFR-PEEK 30/60%, GFR-PEEK 60%, and HA-PEEK 60% had negligible impacts. GFR-PEEK 30%, HA-PEEK 30%, and soft polymers increased cortical stresses and strains by up to 20.5% and 64.5%, respectively. Under axial rotation, titanium ISPs generated tensile stresses of 28.38 MPa at L4 and 17.33 MPa at L5, with corresponding strains of 2049 µε and 1893 µε. While alumina devices slightly decreased these values, soft polymers increased L5 cortical strain beyond 3000 µε, suggesting a heightened risk of tissue overload. Figure 11 illustrates the distribution of maximum principal stresses on the cortical bones of the L4 and L5 segments via zirconia and PEEK ISPs in normal spines, under flexion.

For normal cancellous bone under flexion, titanium ISPs induced tensile stresses ranging from 0.27 to 0.54 MPa and compressive stresses ranging from 1.24 to 1.63 MPa, with peak strains reaching 3516 µε. The ceramic and CFR-PEEK 60% ISPs slightly reduced these values (≤ 6%), maintaining strains below 3440 µε. Other polymeric ISPs increased stresses by up to 25%, increasing strains to 3710 µε. During lateral bending, tensile and compressive stresses with titanium ISPs reached 0.74 MPa and 1.06 MPa, respectively; ceramic devices provided modest reductions, whereas PEEK-based materials slightly increased the values. Under axial rotation, the cancellous stresses ranged from 0.36 to 0.58 MPa at L4 and 0.44 to 0.55 MPa at L5. The strain values remained below 1273 µε (L4) and 1137 µε (L5) with titanium and ceramic ISPs, whereas the strains of the polymer-based devices modestly increased to 1295 µε (L4) and 1226 µε (L5) (Fig. 10).

In the osteoporotic spine model, due to the decrease in vertebral density, the stresses on cancellous bones decrease, increasing the strains on cortical and cancellous bones, compared to the normal spine. However, because the microstructural characteristics of osteoporotic bone (e.g., trabecular thinning, connectivity loss, and increased porosity) were not explicitly modeled, the local stress and strain distribution near the implant–bone interface, and failure mechanisms may be not accurately estimated.

Under flexion with a titanium ISP, osteoporotic cortical bone stresses reached 27.86 MPa (tensile) and 18.19 MPa (compressive) at L4 and 20.00 MPa and 30.74 MPa at L5. The corresponding peak strains were 2231 µε at L4 and 1911 µε at L5. Ceramic ISPs reduced these values by up to 4%, whereas CFR-PEEK 60% had a minimal effect. CFR-PEEK 30%, GFR-PEEK (30% and 60%), and HA-PEEK (30% and 60%) increased both stress and strain by up to 6.5%, with PEEK showing up to a 14% increase relative to titanium (Fig. 12).

During lateral bending, titanium-induced cortical stresses ranged from 14.36 to 26.46 MPa (L4) and 22.60 to 28.42 MPa (L5), with strains approaching 3027 µε. Alumina ISPs reduced these values by up to 26%, whereas CFR-PEEK 60% produced modest reductions. CFR-PEEK 30%, GFR-PEEK, and soft polymers significantly increased the strain, with the compressive strain at L5 surpassing the 3000 µε safety threshold. In axial rotation, titanium ISPs induced stresses of 10.24 to 30.39 MPa (L4) and 11.40 to 16.37 MPa (L5), with compressive strains of up to 2590 µε. The ceramic and CFR-PEEK 60% devices decreased these values to below 2174 µε, whereas soft polymers increased cortical strains above 3000 µε at L5, suggesting elevated fracture risk.

In osteoporotic cancellous bone under flexion, titanium ISPs produced tensile stresses between 0.55 and 0.92 MPa at L4 and between 0.12 and 0.72 MPa at L5, with peak strains of 6574 µε and 6913 µε, respectively. During bending and rotation, the strains ranged from approximately 2300 to 3600 µε in both vertebrae. Ceramic-based ISPs (e.g., alumina, zirconia, ZTA, ATZ) effectively reduce stresses and maintain strains below 6800 µε in flexion, 3542 µε in bending, and 3533 µε in rotation. In contrast, most polymeric devices, except CFR–PEEK 60%, increased stresses and raised strain values beyond the 7000 µε physiological safety limit at L5 during flexion (Fig. 12).

##### Range of motion (ROM) of L4-L5 vertebrae using ISPs

**Fig. 13:**
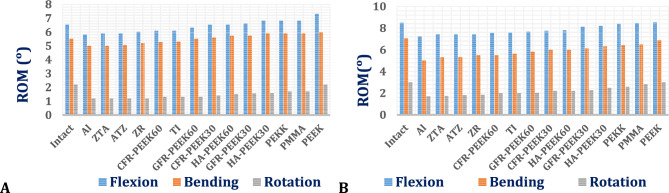
Range of motion in the L4–L5 vertebrae by using ISP with various materials: (A) Normal spine and (**B**) Osteoporotic spine.

Figure 13 presents the range of motion (ROM) at the L4–L5 segment for interspinous process devices (ISPs) fabricated from various materials, evaluated under flexion, lateral bending, and axial rotation in both normal and osteoporotic spine models. A distinct inverse relationship between ISP stiffness and ROM was observed, with higher-stiffness ceramic devices (e.g., alumina) demonstrating lower ROM values and lower-stiffness polymeric devices (e.g., PEEK) exhibiting higher ROM. The ROM was consistently elevated in osteoporotic spines relative to normal spines, particularly under flexion and lateral bending loading conditions.

Notwithstanding these variations, the ROM values for all ISPs remained within physiologically acceptable limits, not exceeding 7° in the normal spine model and 9° in the osteoporotic model.

#### Disc degeneration treatment with the stand-alone ALIF spacer system

##### Stresses on ALIFs

**Fig. 14:**
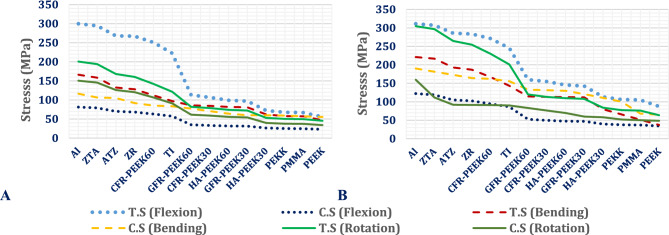
Maximum tensile (T.s.) and compressive (C.S.) stresses on ALIF spacers with various materials in A) Normal spines and B) Osteoporotic spines.

**Fig. 15:**
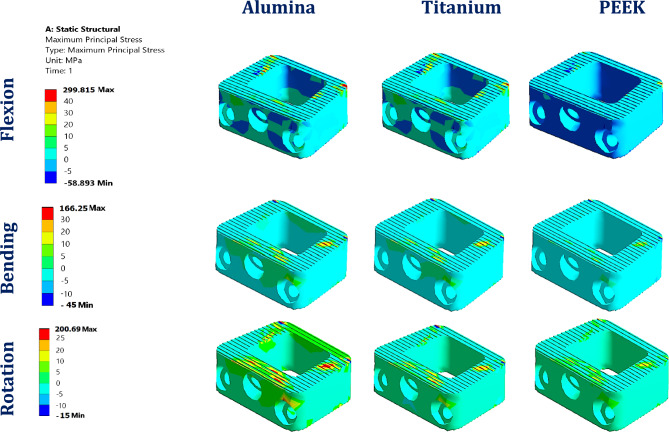
Distribution of maximum principal stresses (MPa) on alumina, titanium, and PEEK ALIFs in a normal spine.

The biomechanical performance of stand-alone anterior lumbar interbody fusion (ALIF) systems fabricated from ceramic and polymeric alternatives to titanium was assessed under flexion, lateral and bending, and axial rotation using both normal and osteoporotic lumbar spine models, as shown in Figs. 14 and 15. High-stiffness materials, including ceramics and carbon fiber-reinforced PEEK with 60% fiber content (CFR-PEEK60%), consistently generated the highest tensile and compressive stress levels. Compared with titanium systems, ALIF systems constructed from alumina exhibited stress increases of up to 35% in normal spines, whereas in the osteoporotic model, the corresponding increase exceeded 26%. Similar trends were observed for zirconia, ZTA, and ATZ-based ALIF devices. Compared with titanium, the CFR-PEEK60% composite also led to moderate stress increases.

In contrast, ALIF cages manufactured from lower-stiffness composites—such as CFR-PEEK30%, GFR-PEEK30%, GFR-PEEK60%, HA-PEEK30%, and HA-PEEK60%—result in marked reductions in both tensile and compressive stresses. These decreases exceeded 49% during flexion in the normal spine and 34% in the osteoporotic spine. The greatest stress reductions were achieved with soft polymeric ALIF systems composed of PEKK, PMMA, and PEEK, which reduced stress magnitudes by more than 56% across both spinal conditions.

**Fig. 16:**
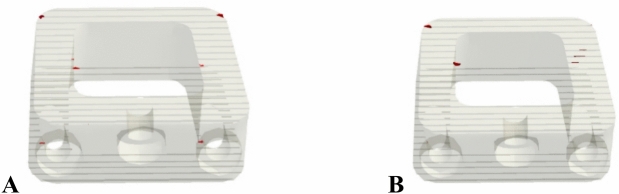
Expected fracture areas (red areas) in alumina ALIF in (**A**) Flexion and (**B**) Rotation.

Stress analysis indicated that the alumina ALIF system exceeded its tensile yield strength (275 MPa) during flexion in the normal spine and during both flexion and rotation in the osteoporotic model, indicating a heightened risk of mechanical failure. The locations of the predicted fracture regions associated with tensile stress concentrations above the yield threshold are highlighted in Fig. 16 in osteoporotic model. Similarly, HA-PEEK30%, HA-PEEK60%, and PMMA ALIF devices presented stress levels surpassing their respective material limits under both conditions, suggesting the potential for structural compromise. In contrast, ALIF systems fabricated from zirconia, ZTA, ATZ, CFR-PEEK30% and 60%, GFR-PEEK30% and 60%, PEKK, and PEEK remained within safe mechanical thresholds under all loading scenarios, reflecting superior durability and safety performance.

##### Stresses and strains on the L4 and L5 segments via ALIFs

**Fig. 17:**
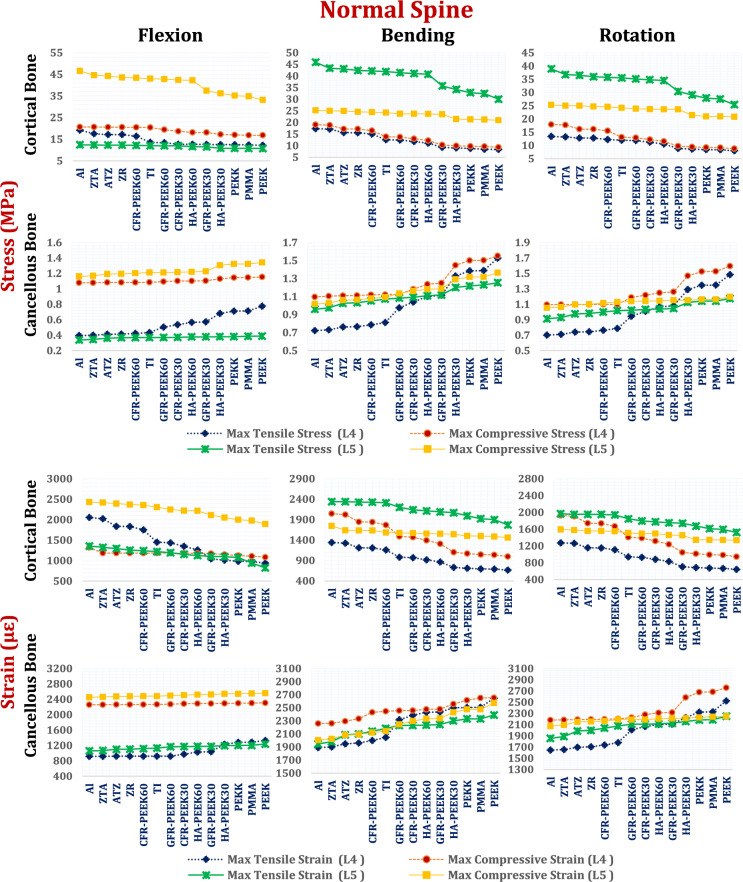
Tensile and compressive stresses (MPa) and strains (µԑ) on the cortical and cancellous bones of the L4 and L5 segments via stand-alone ALIFs in normal spines.

**Fig. 18:**
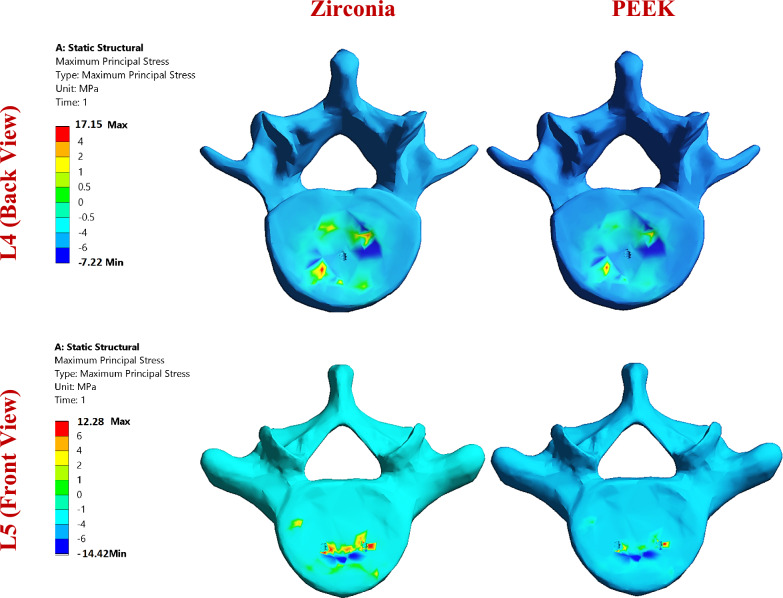
Distribution of maximum principal stresses (MPa) on the cortical bones of the L4 and L5 segments via zirconia and PEEK ALIFs in a normal spine, under flexion.

**Fig. 19:**
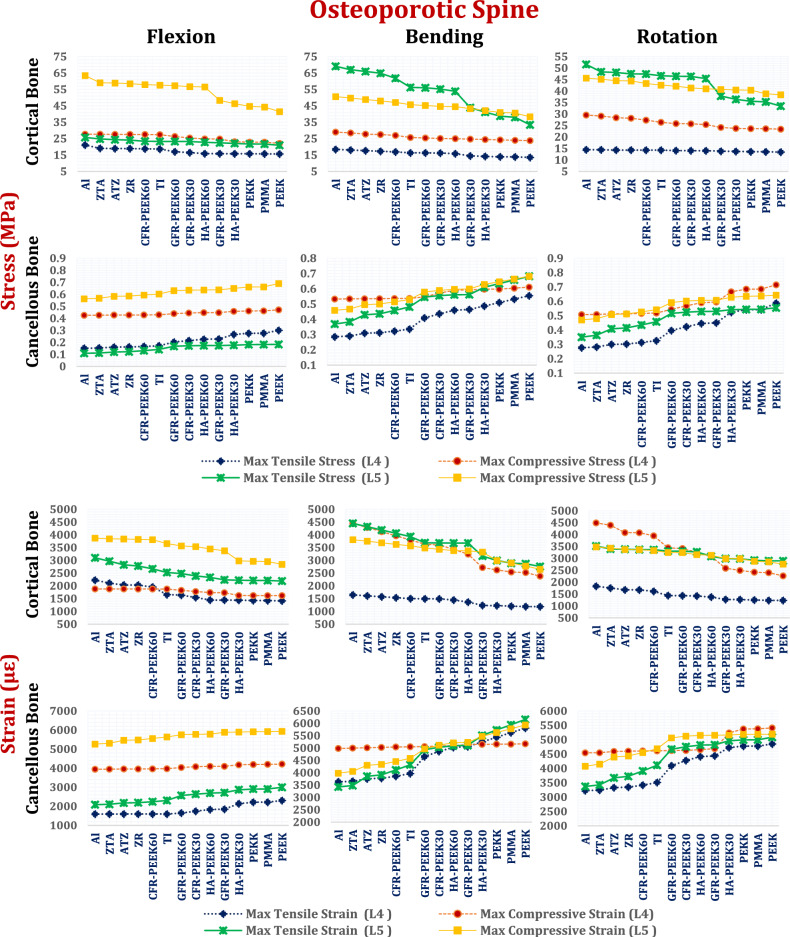
Tensile and compressive stresses (MPa) and strains (µԑ) on the cortical and cancellous bones of the L4 and L5 segments via stand-alone ALIF in normal osteoporotic spines.

The biomechanical response of stand-alone anterior lumbar interbody fusion (ALIF) cages was evaluated using normal and osteoporotic spine models under flexion, lateral bending, and axial rotation. Detailed outcomes are provided in Figs. 17–19, with Fig. 18 illustrating representative distributions of maximum principal stresses on the cortical bone of the L4 and L5 segments for zirconia and PEEK ALIFs in the normal spine model.

In the normal spine (Fig. 17), cortical bone subjected to flexion with a titanium ALIF system presented maximum tensile and compressive stresses of 13.57 MPa and 20.44 MPa on L4 and 12.04 MPa and 43.06 MPa on L5, with associated peak strains of 1452 µε and 2306 µε, respectively. The alumina-based ALIF cages resulted in the greatest increase in stress and strain, with tensile stresses on L4 increasing by up to 41.5%. Other high-stiffness ALIFs—such as those fabricated from ZTA, ATZ, zirconia, and CFR-PEEK60%—also increased the tensile stresses on L4 by more than 20%, although the compressive stress changes were modest. In contrast, the GFR-PEEK60%, CFR-PEEK30%, GFR-PEEK30%, HA-PEEK30%, and HA-PEEK60% cages reduced cortical bone stresses and strains, compared to titanium, whereas the soft polymeric ALIFs (PEKK, PMMA, and PEEK) yielded the lowest mechanical loads, reducing strains to below 1125 µε on L4 and 2000 µε on L5. Similar trends were observed during bending and rotation, with ceramic and CFR-PEEK ALIFs increasing cortical bone stress by up to 38%, whereas polymeric composites decreased it by as much as 34%.

Cancellous bone in the normal spine showed maximum tensile and compressive stresses of 0.433 MPa and 1.08 MPa, respectively, on L4 and 0.369 MPa and 1.211 MPa, respectively, on L5, with corresponding strains of up to 2482 µε. High-stiffness ALIFs such as ceramics and CFR-PEEK60% reduced these stresses by up to 9%, maintaining strains below 2500 µε. In contrast, softer polymers increased cancellous bone loading, although values remained within safe limits, with maximum stress and strain not exceeding 1.342 MPa and 2560 µε, respectively. During bending, titanium ALIF systems produce stresses ranging from 0.812 to 1.122 MPa, with strains reaching 2445 µε. Ceramic-based ALIFs reduced these values by up to 11%, whereas softer polymers increased them, peaking at 1.55 MPa and 2654 µε. Comparable patterns were observed under rotation, with ceramic ALIFs moderately lowering stresses and strains and polymeric ALIFs (excluding CFR-PEEK60%) increasing values, although all remained within physiological safety margins.

In the osteoporotic spine model (Fig. 19), cortical bone exhibited markedly elevated mechanical responses. The titanium ALIF systems generated peak tensile and compressive stresses of 23.30 MPa and 57.6 MPa, respectively, on L5 during flexion, with compressive strains exceeding 3000 µε—surpassing safe thresholds and indicating high fracture risk. Ceramic and CFR-PEEK60% ALIFs further increased these stresses by up to 13%, driving compressive strains on L5 above 3000 µε and increasing the probability of failure. Intermediate-stiffness composites such as GFR-PEEK30%, GFR-PEEK60%, HA-PEEK60%, and CFR-PEEK30% lowered stresses but did not reduce strains below the safety threshold. In contrast, soft polymeric ALIFs (HA-PEEK30%, PEKK, PMMA, and PEEK) significantly reduced cortical bone stresses and successfully maintained strain values below 3000 µε, effectively mitigating fracture risk. These patterns persisted across all loading modes, with L5 being especially susceptible to overload under stiffer cage conditions.

In osteoporotic cancellous bone, titanium ALIFs produced tensile and compressive stresses ranging from 0.141 to 0.603 MPa across L4 and L5 under all loading scenarios. The corresponding strains were highest during flexion, reaching 5642 µε at L5, with bending- and rotation-inducing strains above 4000 µε. Ceramic and CFR-PEEK60% ALIFs effectively reduced these values, maintaining strain levels below 5560 µε (flexion), 5050 µε (bending), and 4610 µε (rotation). While polymeric ALIFs (except CFR-PEEK60%) increased cancellous bone stresses, strains remained below the 7000 µε safety threshold, confirming their biomechanical safety despite elevated loading.

##### Range of motion via ALIFs

**Fig. 20:**
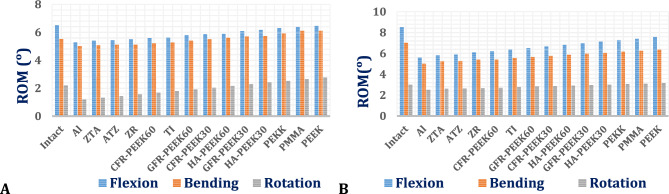
Range of motion in the L4–L5 segments by using standalone ALIF with unconventional materials in (**A**) Normal spines and (**B**) Osteoporotic spines.

The range of motion (ROM) of standalone ALIF systems fabricated from various materials was evaluated in both normal and osteoporotic lumbar spine models under flexion, lateral bending, and axial rotation. As illustrated in Fig. 20, the stiffness of the ALIF system significantly influenced the ROM, with softer polymeric constructs showing the greatest ROM values. Across all tested ALIF systems, the ROM remained within physiological limits, not exceeding 7° in the normal model and 9° in the osteoporotic model.

### Performance evaluation of AI-based regression models for predicting the biomechanical responses of spinal devices with different materials

**Fig. 21:**
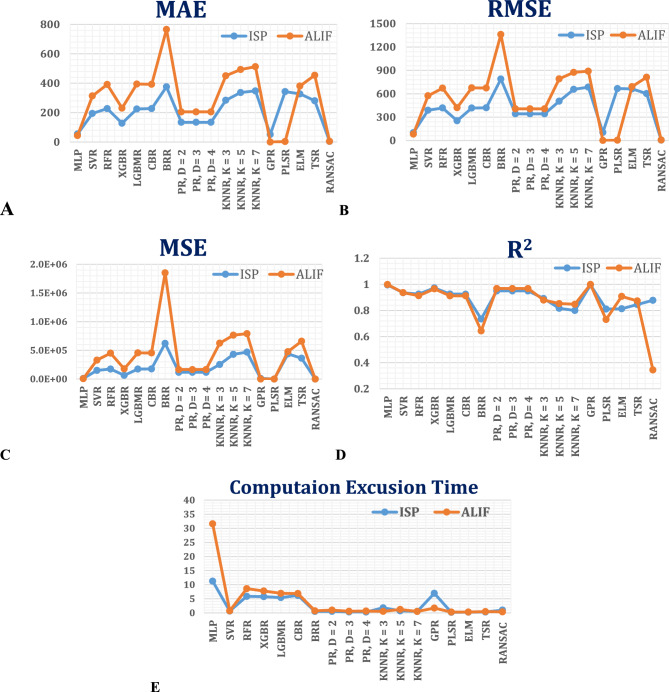
Comparative evaluation of regression models for predicting the biomechanical responses of ALIFs and ISPs under different movements: (**A**) Mean absolute error (MAE), (**B**) Root mean squared error (RMSE), (**C**) Mean squared error (MSE), (**D**) Accuracy (R^2^), and (**E**) Execution time.

The second phase of this study involved a comprehensive evaluation of regression-based machine learning and deep learning models to predict key biomechanical parameters—namely, stress, strain, and ROM—of the vertebral L4–L5 segments fitted with ALIF cages and ISP devices simulated with various materials under different loading conditions. These parameters were derived from FEM simulations and employed as training data for a broad spectrum of regression algorithms. Model performance was assessed via key statistical metrics: the MAE, MSE, RMSE, R^2^, and computational execution time (see Table S2 and Table S3). The performance comparisons are summarized in Fig. 21.

Among the models evaluated, Gaussian process regression (GPR) consistently showed superior predictive accuracy and robustness across both the ALIF and ISP datasets, achieving near-perfect R^2^ values (0.9993 for ALIF and 0.9954 for ISP) with low RMSE values, albeit with moderate inference times (~ 7 s). The multilayer perceptron (MLP) also demonstrated strong predictive performance but needed substantially longer execution times because of its complex architecture, limiting its applicability in time-sensitive scenarios. The extreme learning machine (ELM) provided rapid predictions (< 0.3 s) but at a notable cost to accuracy. Among the ensemble approaches, XGBoost regression (XGBR) achieves a favorable balance between accuracy and computational efficiency, outperforming the LightGBM, CatBoost, and random forest models, which exhibit moderate performance coupled with longer runtimes. Support vector regression (SVR) and polynomial regression models displayed limited flexibility and reduced predictive accuracy, particularly on the more complex ALIF dataset. Simpler algorithms such as K-nearest neighbors (KNN) and partial least squares regression (PLSR) underperformed because of inadequate generalizability and the inability to capture nonlinear biomechanical relationships. Robust regression methods, including RANSAC and Theil–Sen estimators, yielded inconsistent and unreliable predictions.

Overall, advanced nonlinear models—namely, GPR, MLP, and XGBR—most effectively captured the complex biomechanical responses, particularly for anatomically intricate ALIF spacers, whereas the simpler geometries in the ISP dataset eased higher predictive accuracy across a broader range of model.

## Discussion

In the present study, finite element analysis (FEA) served as the primary method to rigorously evaluate the biomechanical performance of two devices—anterior lumbar interbody fusion (ALIF) cages and interspinous process (ISP) devices—at the L4–L5 spinal segment. The analysis used a wide range of materials (zirconia, alumina, ZTA, ATZ, PEKK, PEEK, PMMA, CFR-PEEK, GFR-PEEK, and HA-PEEK), loading conditions (flexion, bending, and rotation), and bone qualities (normal and osteoporotic). Artificial intelligence (AI) was applied not as a substitute for FEA, but as a complementary tool for rapid prediction of biomechanical outcomes based on material and patient-specific parameters. This combined FEA–AI approach has the potential to shorten research and development cycles for spinal devices and to enable real-time, data-informed decision-making in clinical settings.

Several of the materials evaluated in this study are already FDA-approved or CE-marked for use in spinal implants. For example, PEEK and CFR-PEEK are commonly used in interbody cages and interspinous process (ISP) devices^29–33,38,63^. PMMA is widely employed in vertebroplasty and kyphoplasty procedures due to its established clinical safety^34,35^. Ceramic materials like zirconia-toughened alumina (ZTA) and alumina are also approved for use in orthopedic applications, and ZTA has shown potential in experimental spinal implant studies^24,25,27–29,60–62^. In contrast, materials such as HA-PEEK, GFR-PEEK, and ceramic composites are not yet broadly approved for spinal use but have demonstrated promising in vitro biocompatibility and mechanical performance.

Incorporating osteoporotic bone models into finite element analysis provides clinically relevant insights, particularly for cases of mild to moderate osteoporosis where spinal device implantation is still viable^1,3,4,7,54^. Such simulations enable preoperative planning by revealing potential failure modes, safety margins, and stress concentrations, aiding surgeons in risk–benefit assessments for patients with borderline bone quality. They also support device optimization for high-risk populations by identifying materials and configurations that perform better in low-density bone. In this study, including both normal and osteoporotic conditions ensured a more complete biomechanical evaluation, addressing the needs of aging populations with a high prevalence of osteoporosis.

In the first phase of the present study, validation of the FEM spine model was performed by comparing predicted ROM values to those reported in experimental and computational studies^87–95^. The model’s behavior was shown to be within acceptable ranges. Sensitivity analysis revealed that variations in parameters such as moment magnitude, disc stiffness, and facet joint friction had limited influence on outputs, with changes in ROM and von Mises stress remaining under 7%. These findings confirm that the model was robust under a range of physiologically relevant conditions and were consistent with previous reports emphasizing the importance of boundary and contact definitions. The hypothesis that certain non-metallic materials could match or exceed the performance of titanium and that AI could be used to accurately predict mechanical responses was partially supported.

In lumbar segment rehabilitation using interspinous process devices (ISPs), device strength and failure risk were evaluated by comparing induced stresses to material yield strengths. The stress levels for most ISPs remain within acceptable limits; however, the hydroxyapatite PEEK(HA-PEEK) device has shown potential for failure in osteoporotic models due to elevated tensile stresses. Ceramic and carbon fiber-reinforced PEEK with 60% fiber content (CFR-PEEK 60%) ISPs outperformed titanium devices in reducing stress on the L4 and L5 vertebrae, whereas softer polymeric ISPs significantly increased stress levels. In normal bone density models, polymeric ISPs increase the risk of vertebral failure, and in osteoporotic models, strains often exceed safety thresholds, indicating a heightened failure risk. Soft polymeric ISPs also increased vertebral range of motion (ROM), with osteoporotic vertebrae exhibiting greater mobility. Consequently, stiff ceramic and CFR-PEEK 60% materials have been recommended for ISPs in both normal and osteoporotic spines. These findings are in agreement with previous studies in which stiff devices (e.g. metals) were shown to offer sufficient segmental stabilization^11,12,64,96–100^. However, the usage of soft polymeric material (e.g. PEEK) in ISPs has been limited by its reduced mechanical strength^31,32^.

For lumbar rehabilitation employing anterior lumbar interbody fusion (ALIF) spacers, the ceramic and CFR-PEEK 60% cages presented the highest tensile and compressive stresses compared with titanium, whereas the other polymeric ALIF spacers presented the lowest stress levels. Certain materials—including alumina, HA-PEEK (30% and 60%), and PMMA—surpassed their yield strengths under loading in both normal and osteoporotic models, indicating potential structural failure. The ceramic and CFR-PEEK 60% ALIF cages increased cortical bone stress but reduced stress in the cancellous bone of the L4 and L5 vertebrae, whereas polymeric ALIFs had the opposite effect. The stiffness of the ALIF cage influenced the ROM, with softer polymeric devices producing the greatest ROM, yet never exceeding 9° in either bone density model. Therefore, soft polymers such as PEEK and PEKK have been recommended for ALIF spacers under these spinal conditions. These findings are in agreement with previous studies in which PEEK material has been recommended in fusion cages and spacers due to its favorable balance between mechanical strength and shock absorbing ability^15,17,31,32,101–104^. Moreover, Chatham et al*.*^102^, via finite element analysis of the L4–L5 segment, demonstrated that PEEK spacers reduced stress concentrations on vertebral endplates, potentially mitigating the risk of cage subsidence.

In the second phase of the present study, regression-based machine learning and deep learning models were employed to predict stress, strain, and ROM for the L4-L5 vertebra using ALIF cages and ISP devices under various loading and material conditions, in normal and osteoporotic spines. Among the models tested, Gaussian Process Regression (GPR) and Multilayer Perceptron (MLP) were found to provide the most accurate predictions. Ensemble learning models, such as XGBoost and LightGBM, also demonstrated good performance while offering faster computation, making them suitable for clinical support systems requiring rapid estimations. The integration of FEM with AI modeling was shown to offer a powerful tool for device screening, material optimization, and pre-surgical planning, especially when patient-specific geometries and material data are available. This hybrid approach allows for biomechanical behavior to be predicted efficiently, without the need for extensive computational resources.

A key limitation of this study was the representation of osteoporotic bone solely through a reduction in elastic modulus, consistent with previous FE studies^54,105,106^. While this approach captures the overall reduction in bone stiffness, it does not account for microstructural changes, such as trabecular thinning, cortical porosity, or region-specific bone loss, that are commonly observed in clinical osteoporosis. Consequently, the model may inaccurately estimate local stress concentrations, micro-fracture risk, and implant micromotion in patients with severe bone loss, potentially leading to an under- or overestimation of bone failure, implant performance, fixation stability, and the likelihood of mechanical failure under osteoporotic conditions.

Additionally, all device materials were modeled as linear elastic and isotropic, with failure risk assessed by comparing principal stresses to uniaxial yield strengths. While this approach allows for consistent comparisons across different materials, it neglects important behaviors such as the brittleness and fracture toughness of ceramics, the anisotropy and progressive damage mechanisms of fiber-reinforced composites, and the viscoelastic, pressure-sensitive response of polymers. As a result, the absolute stress predictions may not fully represent long-term implant performance, time-dependent phenomena such as fatigue, creep, and stress relaxation, or the initiation and progression of material-specific failure modes under physiological loading.

A further limitation was that the analysis was restricted to a single spinal level (L4–L5), which may reduce the generalizability of the findings to other spinal regions that differ in anatomical geometry, kinematics, and load-sharing characteristics. Multilevel constructs, adjacent-segment interactions, and compensatory motions were not included, meaning the results should be interpreted as representative of a controlled single-level scenario rather than the entire spine under physiological loading conditions.

Future research should address these limitations by incorporating nonlinear and time-dependent material models, as well as fatigue and progressive damage analyses, to improve the prediction of long-term implant performance. Patient-specific probabilistic modeling could better capture inter-individual variability and provide a more accurate assessment of mechanical failure risk across diverse patient populations. Furthermore, micro-CT–based geometries or multiscale modeling approaches should be employed to account for microstructural bone alterations, such as trabecular thinning, loss of connectivity, and increased porosity, that are characteristic of osteoporosis. Extending the simulations to multiple spinal levels and integrating patient-specific anatomies derived from imaging data would further enhance clinical relevance and improve the predictive accuracy of implant performance under real-world physiological conditions.

## Conclusion

The primary objective of this study was to evaluate ceramics, composites, and polymers as alternatives to titanium in the production of two anterior and posterior spinal devices, ALIF and ISP, using the finite element method (FEM) and artificial intelligence (AI). Finite element method (FEM) simulations on both normal and osteoporotic spinal models were used to analyze the mechanical responses, including the stress and strain distributions, and range of motion (ROM), of various device materials. Additionally, advanced artificial intelligence (AI) regression models were developed to predict FEM-derived mechanical outcomes, enabling rapid assessment of device performance across different material properties.

Within the scope of single-level L4–L5 finite element analysis under simulated physiological conditions, the use of high-stiffness materials—such as ceramics and 60% carbon fiber–reinforced PEEK (CFR-PEEK 60%)—in interspinous process (ISP) devices was associated with a moderate reduction in stress and strain transmission to adjacent vertebral segments, potentially mitigating the risk of bone failure. This increased stiffness, however, also resulted in a measurable reduction in the segmental range of motion (ROM). Conversely, for stand-alone ALIF devices, lower-stiffness polymers exhibited improved modulus compatibility with cortical bone, thereby decreasing cortical stress concentrations. Nevertheless, these materials produced elevated stresses and strains in cancellous bone—remaining below reported failure thresholds—attributable to greater deformation and load transfer, which in turn led to increased ROM at the operated level.

The findings for osteoporotic conditions should be viewed as indicative of overall biomechanical trends rather than definitive clinical outcomes. Clinical decision-making should incorporate patient-specific imaging, bone quality evaluation, and surgical assessment rather than relying solely on computational predictions.

## Clinical and research implications

The single-level L4–L5 finite element analysis, performed under simulated normal and osteoporotic bone conditions, yields the following key insights:

### Material selection for spinal devices should be individualized, considering

- Bone quality, particularly distinguishing between normal and osteoporotic conditions
- Patient-specific anatomical features, including vertebral geometry and spinal curvature
- Clinical and surgical objectives, such as motion preservation, fusion, or decompression

### High-stiffness materials (e.g., ceramics and CFR-PEEK 60%) may offer

- Superior suitability for interspinous process (ISP) devices
- Enhanced performance in osteoporotic spines, where stability is critical
- A capacity to reduce local strain concentrations
- Effective limitation of the excessive range of motion at the instrumented segment

### Lower-stiffness polymeric materials (e.g., PEEK, PEKK) are more preferable for

- Anterior lumbar interbody fusion (ALIF) cages, where stress distribution is key
- Facilitating load sharing between the cage and the vertebrae
- Promoting uniform stress distribution across endplates, potentially minimizing the risk of failure

### Artificial intelligence (AI)-based models demonstrate

- High predictive accuracy for mechanical outcomes, including stress, strain, and ROM
- Strong potential as clinical decision-support tools to guide:
- Device material selection
- Personalized device design
- A foundation for a data-driven approach to optimize spinal surgery outcomes

### Caution in interpretation

The implications of this study are based on a single-segment (L4–L5) model and do not account for multi-level spinal pathology, obesity, or other complex clinical scenarios. Further research incorporating multi-level models, diverse patient morphologies, and in vivo validation is needed before broader clinical generalization. Additionally, the inclusion of osteoporotic bone conditions in this study is not intended to suggest that device placement is universally advisable for all patients with osteoporosis. In this study, osteoporosis was simulated solely by reducing the elastic modulus of bone tissue, without modeling microstructural alterations, which may limit the accuracy of predicted bone–implant interface mechanics and potential failure modes. In severe cases, markedly reduced bone quality may preclude safe fixation, and alternative treatment options should be prioritized. The osteoporotic simulations presented in this study are intended to provide biomechanical benchmarks to guide the selection of more suitable materials in scenarios where surgical intervention is clinically warranted.

## Supplementary Information

Supplementary Information.

## Data Availability

The datasets generated during and/or analyzed during the current study are available from the corresponding author upon reasonable request.

## Abbreviations

ALIF: Anterior lumbar interbody fusion
ISP: Interspinous process
ZTA: Zirconia-toughened alumina
ATZ: Alumina-toughened zirconia
PEKK: Polyetherketoneketone
PEEK: Polyetheretherketone
PMMA: Polymethylmethacrylate
CFR-PEEK: Carbon fiber-reinforced PEEK
GFR-PEEK: Glass fiber-reinforced PEEK
HA-PEEK: Hydroxyapatite-reinforced PEEK
ROM: Range of motion
MAE: Mean absolute error
MSE: Mean squared error
RMSE: Root mean squared error
R^2^: Coefficient of determination
MLP: Multi-layer perceptron
SVR: Support vector regression
RFR: Random forest regression
BRR: Bayesian ridge regression
PR: Polynomial regression
KNN: K-nearest neighbors
GPR: Gaussian process regression
PLSR: Partial least squares regression
ELM: Extreme learning machine
XGBR: XGBoost regression
LGMR: LightGBM regression
CBR: CatBoost regression
TSR: Theil–Sen estimator regression

## References

[1] Manfrè, L. *Spinal Canal Stenosis* (Springer, 2016).

[2] Walter KL, O’Toole JE. Lumbar Spinal Stenosis. *JAMA.* 328(3):310. 10.1001/jama.2022.6137 10.1001/jama.2022.6137 35503646

[3] Katz, J. N., Zimmerman, Z. E., Mass, H. & Makhni, M. C. Diagnosis and management of lumbar spinal stenosis: A review. *JAMA* **327**(17), 1688–1699. 10.1001/jama.2022.5921 (2022). 35503342 10.1001/jama.2022.5921

[4] Mobbs, R. J., Li, J., Sivabalan, P., Raley, D. & Rao, P. J. Outcomes after decompressive laminectomy for lumbar spinal stenosis: Comparison between minimally invasive unilateral laminectomy for bilateral decompression and open laminectomy. *J. Neurosurg. Spine* **21**(2), 179–186. 10.3171/2014.4.spine13420 (2014). 24878273 10.3171/2014.4.SPINE13420

[5] Gunzburg, R. & Szpalski, M. *Spondylolysis, Spondylolisthesis, and Degenerative Spondylolisthesis* (Lippincott Williams & Wilkins, 2006).

[6] He, D., Li, Z., Zhang, T., Cheng, X. & Tian, W. Prevalence of lumbar spondylolisthesis in middle-aged people in Beijing community. *Orthop. Surg.* **13**(1), 202–206. 10.1111/os.12871 (2021). 33438343 10.1111/os.12871 PMC7862163

[7] Pinheiro-Franco, J. L., Vaccaro, A. R., Benzel, E. C. & Mayer, H. M. *Advanced Concepts in Lumbar Degenerative Disk Disease* (Springer, 2015).

[8] My Vanderbilt Health. (2025). *Degenerative disc disease is more common than you think*. https://my.vanderbilthealth.com/lumbar-degenerative-disc-disease-is-more-common-than-you-think/

[9] Ravindra, V. M. et al. Degenerative lumbar spine disease: Estimating global incidence and worldwide volume. *Glob. Spine J.* **8**(8), 784–794. 10.1177/2192568218770769 (2018). 10.1177/2192568218770769 PMC6293435 30560029

[10] Moojen, W. A. et al. Interspinous process device versus standard conventional surgical decompression for lumbar spinal stenosis: randomized controlled trial. *BMJ* **347**, 6415. 10.1136/bmj.f6415 (2013). 10.1136/bmj.f6415 PMC3898636 24231273

[11] Paradigm Spine GmbH. (2023). Coflex® *Interlaminar Stabilization® Paradigm Spine GmbH.* https://paradigmspine.com/en_gb/product/coflex-interlaminar-stabilization/

[12] Davis, R. J., Errico, T. J., Bae, H. & Auerbach, J. D. Decompression and coflex interlaminar stabilization compared with decompression and instrumented spinal fusion for spinal stenosis and Low-Grade degenerative spondylolisthesis. *Spine* **38**(18), 1529–1539. 10.1097/brs.0b013e31829a6d0a (2013). 23680830 10.1097/BRS.0b013e31829a6d0a

[13] Pei, B. et al. Biomechanical comparative analysis of conventional pedicle screws and cortical bone trajectory fixation in the lumbar spine: An in vitro and finite element study. *Front. Bioeng. Biotechnol.*10.3389/fbioe.2023.1060059 (2023). 36741751 10.3389/fbioe.2023.1060059 PMC9892841

[14] Liu, Z., Zhang, S., Li, J. & Tang, H. Biomechanical comparison of different interspinous process devices in the treatment of lumbar spinal stenosis: a finite element analysis. *BMC Musculoskelet. Disord.*10.1186/s12891-022-05543-y (2022). 35715775 10.1186/s12891-022-05543-y PMC9204899

[15] Burkus, J. K., Gornet, M. F., Dickman, C. A. & Zdeblick, T. A. Anterior lumbar interbody fusion using RHBMP-2 with tapered interbody cages. *J. Spinal Disord. Tech.* **15**(5), 337–349. 10.1097/00024720-200210000-00001 (2002). 12394656 10.1097/00024720-200210000-00001

[16] *ALIF Surgery: Anterior lumbar interbody Fusion | HSS Spine*. (n.d.). Hospital for Special Surgery. https://www.hss.edu/conditions_alif-anterior-lumbar-interbody-fusion-spine-surgery.asp#:~:text=Anterior%20lumbar%20interbody%20fusion%20(ALIF,in%20the%20lower%20back%20together.

[17] Guyer, R. D., Zigler, J. E., Blumenthal, S. L., Shellock, J. L. & Ohnmeiss, D. D. Evaluation of anterior lumbar interbody fusion performed using a stand-alone, integrated fusion cage. *Int. J. Spine Surg.* **17**(1), 1–5. 10.14444/8354 (2022). 35940637 10.14444/8354 PMC10025836

[18] Mumtaz, M., Zafarparandeh, I. & Erbulut, D. U. Investigation into cervical spine biomechanics following single, multilevel and hybrid disc replacement surgery with dynamic cervical implant and fusion: A finite element study. *Bioengineering* **9**(1), 16. 10.3390/bioengineering9010016 (2022). 35049725 10.3390/bioengineering9010016 PMC8773264

[19] Mo, Z. J. et al. Biomechanical effects of cervical arthroplasty with U-shaped disc implant on segmental range of motion and loading of surrounding soft tissue. *Eur. Spine J.* **23**(3), 613–621. 10.1007/s00586-013-3070-4 (2013). 24154828 10.1007/s00586-013-3070-4 PMC3940800

[20] Shayesteh Moghaddam, N. et al. Metals for bone implants: Safety, design, and effcacy. *Biomanuf. Rev.* **1**, 1–16 (2016).

[21] Saha, S. & Roy, S. Metallic dental implants wear mechanisms, materials, and manufacturing processes: A literature review. *Materials* **16**(1), 161 (2022). 36614500 10.3390/ma16010161 PMC9821388

[22] Rojas, A. R., Elguezabal, A. A., Porporati, A. A., Bernal, M. B. & Ponce, H. E. E. Metals and alloys choice for implants. *In Synthesis lectures on biomedical engineering* (pp. 23–48) (2023). 10.1007/978-3-031-25420-8_4

[23] Kölle, L., Ignasiak, D., Ferguson, S. & Helgason, B. Ceramics in total disc replacements: A scoping review. *Clin. Biomech.*10.1016/j.clinbiomech.2022.105796 (2022). 10.1016/j.clinbiomech.2022.105796 36435073

[24] Hosono, N., Sakaura, H., Ohwada, T., Yonenobu, K. & Yoshikawa, H. Ceramic spine prostheses. *In CRC Press eBooks* (pp. 373–386) (2003). 10.1201/b14227-17

[25] Cordis, C. (2013). *Advanced multifunctional zirconia ceramics for long-lasting implants.* CORDIS | European Commission. https://cordis.europa.eu/project/id/280741/reporting

[26] Hulbert, S. F. (2013). The use of alumina and zirconia in surgical implants. In *Imperial College Press* eBooks (pp. 27–47). 10.1142/9781908977168_0002

[27] Pobloth, A. et al. Bioactive coating of zirconia toughened alumina ceramic implants improves cancellous osseointegration. *Sci. Rep.*10.1038/s41598-019-53094-5 (2019). 31723174 10.1038/s41598-019-53094-5 PMC6853946

[28] Schierano, G. et al. An alumina toughened zirconia composite for dental implant application: In vivoanimal results. . *BioMed Res. Int.* **2**, 1–9. 10.1155/2015/157360 (2015). 10.1155/2015/157360 PMC4402487 25945324

[29] Warburton, A., Girdler, S. J., Mikhail, C. M., Ahn, A. & Cho, S. K. Biomaterials in spinal implants: A review. *Neurospine* **17**(1), 101–110. 10.14245/ns.1938296.148 (2020). 31694360 10.14245/ns.1938296.148 PMC7136103

[30] Dawson, J. H., Hyde, B., Hurst, M., Harris, B. T. & Lin, W. Polyetherketoneketone (PEKK), a framework material for complete fixed and removable dental prostheses: A clinical report. *J. Prosthet. Dent.* **119**(6), 867–872. 10.1016/j.prosdent.2017.09.008 (2017). 29195815 10.1016/j.prosdent.2017.09.008

[31] Yuan, B. et al. Comparison of osteointegration property between PEKK and PEEK: Effects of surface structure and chemistry. *Biomaterials* **170**, 116–126. 10.1016/j.biomaterials.2018.04.014 (2018). 29660634 10.1016/j.biomaterials.2018.04.014

[32] Senra, M. R., Marques, M. F. V. & Monteiro, S. N. Poly (ether-ether-ketone) for biomedical applications: from enhancing bioactivity to reinforced-bioactive composites—An (2023). 10.3390/polym15020373 PMC9861117 36679253

[33] Tekin, S., Değer, Y. & Demirci, F. Evaluation of the use of PEEK material in implant-supported fixed restorations by finite element analysis. *Niger. J. Clin. Pract.* **22**(9), 1252. 10.4103/njcp.njcp_144_19 (2019). 31489862 10.4103/njcp.njcp_144_19

[34] Ridwan-Pramana, A. et al. Structural and mechanical implications of PMMA implant shape and interface geometry in cranioplasty—A finite element study. *J. Cranio-Maxillofac. Surg.* **44**(1), 34–44. 10.1016/j.jcms.2015.10.014 (2015). 10.1016/j.jcms.2015.10.014 26646634

[35] Lewin, S., Försth, P. & Persson, C. Low-modulus PMMA has the potential to reduce stresses on endplates after cement discoplasty. *J. Funct. Biomater.* **13**(1), 18. 10.3390/jfb13010018 (2022). 35225981 10.3390/jfb13010018 PMC8883899

[36] Kurtz, S. M. *UHMWPE Biomaterials Handbook: Ultra high molecular weight Polyethylene in total joint Replacement and Medical Devices*. https://www.amazon.com/UHMWPE-Biomaterials-Handbook-Second-Polyethylene/dp/0124015069.

[37] Wo, J. et al. Biomechanical analysis of cervical artificial disc replacement using cervical subtotal discectomy prosthesis. *Front. Bioeng. Biotechnol.*10.3389/fbioe.2021.680769 (2021). 34336799 10.3389/fbioe.2021.680769 PMC8317600

[38] Han, X. et al. Carbon fiber reinforced PEEK composites based on 3D-Printing technology for orthopedic and dental applications. *J. Clin. Med.* **8**(2), 240. 10.3390/jcm8020240 (2019). 30759863 10.3390/jcm8020240 PMC6406436

[39] Ma, H. et al. PEEK (Polyether-ether-ketone) and its composite materials in orthopedic implantation. *Arab. J. Chem.* **14**(3), 102977. 10.1016/j.arabjc.2020.102977 (2021).

[40] Ma, R. & Guo, D. Evaluating the bioactivity of a hydroxyapatite-incorporated polyetheretherketone biocomposite. *J. Orthop. Surg. Res.*10.1186/s13018-019-1069-1 (2019). 30683125 10.1186/s13018-019-1069-1 PMC6347847

[41] Shash, Y. H. Assessment of cranial reconstruction utilizing various implant materials: Finite element study. *J. Mater. Sci. Mater. Med.*10.1007/s10856-024-06816-9 (2024). 39136804 10.1007/s10856-024-06816-9 PMC11322413

[42] Dewi, A. H. & Ana, I. D. The use of hydroxyapatite bone substitute grafting for alveolar ridge preservation, sinus augmentation, and periodontal bone defect: A systematic review. *Heliyon* **4**(10), e00884. 10.1016/j.heliyon.2018.e00884 (2018). 30417149 10.1016/j.heliyon.2018.e00884 PMC6218667

[43] Hastie, T., Tibshirani, R. J. & Friedman, J. The elements of statistical learning: data mining, inference, and prediction (2013). http://catalog.lib.kyushu-u.ac.jp/ja/recordID/1416361

[44] McWalter, E. J. & Majumdar, S. Application of image-based modeling to orthopedic biomechanics. *Curr. Opin. Rheumatol.* **23**(5), 483–490. 10.1097/BOR.0b013e328349acdb (2011).

[45] Dall’Ara, E. & Viceconti, M. Virtual mechanical testing based on finite element models: An overview of recent achievements. *Front. Bioeng. Biotechnol.*10.3389/fbioe.2021.646600 (2021).34178966

[46] Shirazi-Adl, A., Arjmand, N. & Parnianpour, M. Computational biomechanics of the human lumbar spine: Models, validation and applications. *Crit. Rev. Biomed. Eng.* **33**(5), 411–469 (2005).

[47] Eskandari, A., Arjmand, N. & Shirazi-Adl, A. Prediction of spine loads from posture and muscle activity using artificial neural networks. *Med. Eng. Phys.* **57**, 75–83. 10.1016/j.medengphy.2018.04.008 (2018). 29691130 10.1016/j.medengphy.2018.04.008

[48] Jalal, M. et al. FEM-based machine learning models for predicting stress distribution in orthopedic implants. *Biomed. Signal Process. Control*10.1016/j.bspc.2021.102708 (2021).

[49] BodyParts3D/Anatomography: Select parts and Make Embeddable Model of Your Own. (n.d.). Retrieved from https://lifesciencedb.jp/bp3d/?lng=en

[50] Ansys SpaceClaim 3D Modeling Software. https://www.ansys.com/products/3d-design/ansys-spaceclaim

[51] Goplani, P. Fracture strength estimation of L3–L4 intervertebral disc using FEA. *Vibroeng. Procedia* **27**, 67–72. 10.21595/vp.2019.20976 (2019).

[52] Ibarz, E. et al. Instability of the lumbar spine due to disc degeneration. A finite element simulation. *Adv. Biosci. Biotechnol.* **4**(4), 548–556. 10.4236/abb.2013.44072 (2013).

[53] Wei, H., Chuang, S. & Chen, C. Biomechanical evaluation of the lumbar spine by using a new interspinous process device: A finite element analysis. *Appl. Sci.* **11**(21), 10486. 10.3390/app112110486 (2021).

[54] Wang, J., Geng, Z., Ma, X., Zhang, Z. & Miao, J. A comparative analysis of using cage acrossing the vertebral ring apophysis in normal and osteoporotic models under endplate injury: A finite element analysis. *Front. Bioeng. Biotechnol.*10.3389/fbioe.2023.1263751 (2023). 38026854 10.3389/fbioe.2023.1263751 PMC10664026

[55] Simion, G., Eckardt, N., Ullrich, B. W., Senft, C. & Schwarz, F. Bone density of the cervical, thoracic and lumbar spine measured using Hounsfield units of computed tomography—Results of 4350 vertebras. *BMC Musculoskelet. Disord.*10.1186/s12891-024-07324-1 (2024). 38443864 10.1186/s12891-024-07324-1 PMC10916010

[56] Morgan, E. F., Unnikrisnan, G. U. & Hussein, A. I. Bone mechanical properties in healthy and diseased states. *Annu. Rev. Biomed. Eng.* **20**, 119–143. 10.1146/annurev-bioeng-062117-121139 (2018). 29865872 10.1146/annurev-bioeng-062117-121139 PMC6053074

[57] Shash, Y. H. Finite element investigation for improving chest wall reconstruction process using ceramic and polymeric implants. *Sci. Rep.*10.1038/s41598-024-79536-3 (2025). 39788988 10.1038/s41598-024-79536-3 PMC11718210

[58] Shash, Y. H., El-Wakad, M. T., El-Dosoky, M. A. & Dohiem, M. M. Evaluation of stresses on mandible bone and prosthetic parts in fixed prosthesis by utilizing CFR-PEEK, PEKK and PEEK frameworks. *Scientific Rep. ***1**, 13(1) (2023). 10.1038/s41598-023-38288-2 10.1038/s41598-023-38288-2 PMC10352298 37460592

[59] Tekin, S., Cangül, S., Adıgüzel, Ö. & Değer, Y. Areas for use of PEEK material in dentistry. *Int. Dent. Res.* **8**(2), 84–92. 10.5577/intdentres.2018.vol8.no2.6 (2018).

[60] Technical Products, Zirconia (ZrO2)—YTZP material specifications, https://www.technicalproductsinc.com/pdf/Specs/Zirconia%20YTZP%20Specs.pdf

[61] AZoM. (n.d.). Properties: Alumina–aluminum oxide–AL2O3—A refractory ceramic oxide. https://www.azom.com/properties.aspx?ArticleID=52

[62] Properties: alumina as a biomaterial (99.5% alumina). (n.d.). https://www.azom.com/properties.aspx?ArticleID=105

[63] Invibio Biomaterial Solutions, Endolign, Updated (7/2022). www. invibio.com.

[64] Guo, L. & Yin, J. Finite element analysis and design of an interspinous device using topology optimization. *Med. Biol. Eng. Comput.* **57**(1), 89–98. 10.1007/s11517-018-1838-8 (2018). 29981052 10.1007/s11517-018-1838-8

[65] Qua, H.-C., Tan, C.-S., Wong, K.-C., Ho, J.-H., Wang, X., Yap, E.-H., Ooi, J.-B. & Wong, Y.-S. *Applied Engineering Failure Analysis*: *Theory and Practice*. (CRC Press, 2015).

[66] Lin, H. et al. Biomechanical comparison of the K-ROD and Dynesys dynamic spinal fixator systems—A finite element analysis. *Bio-Med. Mater. Eng.* **23**(6), 495–505. 10.3233/bme-130766 (2013). 10.3233/BME-130766 24165552

[67] Frost, H. M. Wolff’s Law and bone’s structural adaptations to mechanical usage: an overview for clinicians. *Angle Orthod.* **64**(3), 175–188. 10.1043/0003-3219(1994)064 (2009). 10.1043/0003-3219(1994)064<0175:WLABSA>2.0.CO;2 8060014

[68] Limbert, G. et al. Trabecular bone strains around a dental implant and associated micromotions—A micro-CT-based three-dimensional finite element study. *J. Biomech.* **43**(7), 1251–1261. 10.1016/j.jbiomech.2010.01.003 (2010). 20170921 10.1016/j.jbiomech.2010.01.003

[69] Taud, H. & Mas, J. (2017). Multilayer Perceptron (MLP). In Lecture notes in geoinformation and cartography (pp. 451–455). 10.1007/978-3-319-60801-3_27

[70] Brereton, R. G. & Lloyd, G. R. Support vector machines for classification and regression. *Analyst* **135**(2), 230–267. 10.1039/b918972f (2009). 20098757 10.1039/b918972f

[71] Breiman, L. Random forests. *Mach. Learn.* **45**(1), 5–32. 10.1023/A:1010933404324 (2001).

[72] Geurts, P., Ernst, D. & Wehenkel, L. Extremely randomized trees. *Mach. Learn.* **63**(1), 3–42. 10.1007/s10994-006-6226-1 (2006).

[73] Chen, T. & Guestrin, C. XGBoost: A scalable tree boosting system. *Proceedings of the 22nd ACM SIGKDD International Conference on Knowledge Discovery and Data Mining* (2016). 10.1145/2939672.2939785

[74] Ke, G., *et al*. Lightgbm: A highly efficient gradient boosting decision tree. *Adv. Neural Inf. Process. Syst.* **30** (2017).

[75] Prokhorenkova, L., Gusev, G., Vorobev, A., Dorogush, A. V. & Gulin, A. CatBoost: Unbiased boosting with categorical features. *Adv. Neural Inf. Process. Syst.* **31** (2018).

[76] Wang, X., Yue, Y. R. & Faraway, J. J. *Bayesian Regression Modeling with INLA* (CRC Press, 2018).

[77] Smola, A. J. & Schölkopf, B. A tutorial on support vector regression. *Stat. Comput.* **14**(3), 199–222. 10.1023/B:STCO.0000035301.49549.88 (2004).

[78] Peterson, L. K-nearest neighbor. *Scholarpedia* **4**(2), 1883. 10.4249/scholarpedia.1883 (2009).

[79] Shi, J. Q. & Choi, T. *Gaussian Process Regression Analysis for Functional Data* (CRC Press, 2011).

[80] Vinzi, V. E., Chin, W. W. & Henseler, J. R. *Handbook of Partial Least Squares* (Springer, 2010).

[81] Sun, F., Toh, K., Romay, M. G. & Mao, K. *Extreme Learning Machines 2013: Algorithms and Applications*. Springer (2014).

[82] Wilcox, R. R. Some results on extensions and modifications of the Theil-Sen regression estimator. *Br. J. Math. Stat. Psychol.* **57**(2), 265–280. 10.1348/0007110042307230 (2004). 15511308 10.1348/0007110042307230

[83] Lopez, U., Trujillo, L., Martinez, Y., Legrand, P., Naredo, E. & Silva, S. RANSAC-GP: Dealing with Outliers in Symbolic Regression with Genetic Programming. In *Lecture notes in computer science* (pp. 114–130) (2017). 10.1007/978-3-319-55696-3_8

[84] Dodge, Y. *The Concise Encyclopedia of Statistics*. Springer Science & Business Media (2008).

[85] Wang, W. *Principles of machine learning: The Three Perspectives*. Springer Nature (2024).

[86] Chai, T. & Draxler, R. R. Root mean square error (RMSE) or mean absolute error (MAE)? Arguments against avoiding RMSE in the literature. *Geosci. Model Dev.* **7**(3), 1247–1250. 10.5194/gmd-7-1247-2014 (2014).

[87] Liu, J. et al. Biomechanical properties of a novel nonfusion artificial vertebral body for anterior lumbar vertebra resection and internal fixation. *Sci. Rep.*10.1038/s41598-021-82086-7 (2021). 33514823 10.1038/s41598-021-82086-7 PMC7846776

[88] Yamamoto, I., Panjabi, M. M., Crisco, T. & Oxland, T. Three-dimensional movements of the whole lumbar spine and lumbosacral joint. *Spine* **14**(11), 1256–1260. 10.1097/00007632-198911000-00020 (1989). 2603060 10.1097/00007632-198911000-00020

[89] Zhang, C., Berven, S. H., Fortin, M. & Weber, M. H. Adjacent segment degeneration versus disease after lumbar spine fusion for degenerative pathology: A systematic review with meta-analysis of the literature. *Clin. Spine Surg.* **29**(1), 21–29 (2016). 26836484 10.1097/BSD.0000000000000328

[90] Panjabi, M. M., Oxland, T. R., Yamamoto, I. & Crisco, J. J. Mechanical behavior of the human lumbar and lumbosacral spine as shown by three-dimensional load-displacement curves. *J. Bone Jt. Surg.* **76**(3), 413–424. 10.2106/00004623-199403000-00012 (1994). 10.2106/00004623-199403000-00012 8126047

[91] Calvo-Echenique, A., Cegoñino, J. & Del Palomar, A. P. Is there any advantage of using stand-alone cages? A numerical approach. . *BioMed. Eng. OnLine* **18**(1), 1. 10.1186/s12938-019-0684-8 (2019). 31113423 10.1186/s12938-019-0684-8 PMC6530002

[92] Rastegar, S., Arnoux, P., Wang, X. & Aubin, C. Biomechanical analysis of segmental lumbar lordosis and risk of cage subsidence with different cage heights and alternative placements in transforaminal lumbar interbody fusion. *Comput. Methods Biomech. Biomed. Eng.* **23**(9), 456–466. 10.1080/10255842.2020.1737027 (2020). 10.1080/10255842.2020.1737027 32169009

[93] Jaramillo, H. E., Puttlitz, C. M., McGilvray, K. & García, J. J. Characterization of the L4–L5–S1 motion segment using the stepwise reduction method. *J. Biomech.* **49**(7), 1248–1254. 10.1016/j.jbiomech.2016.02.050 (2016). 27017302 10.1016/j.jbiomech.2016.02.050

[94] Dahl, M. C., Ellingson, A. M., Mehta, H. P., Huelman, J. H. & Nuckley, D. J. The biomechanics of a multilevel lumbar spine hybrid using nucleus replacement in conjunction with fusion. *Spine J.* **13**(2), 175–183. 10.1016/j.spinee.2012.11.045 (2013). 23318109 10.1016/j.spinee.2012.11.045

[95] Shim, C. S. et al. Biomechanical evaluation of an interspinous stabilizing device, locker. *Spine* **33**(22), E820–E827. 10.1097/brs.0b013e3181894fb1 (2008). 18923305 10.1097/BRS.0b013e3181894fb1

[96] Errico, T. J., Kamerlink, J. R., Quirno, M., Samani, J. & Chomiak, R. J. Survivorship of coflex Interlaminar-Interspinous Implant. *SAS J.* **3**(2), 59–67. 10.1016/s1935-9810(09)70008-8 (2009). 25802629 10.1016/SASJ-2008-0027-RR PMC4365593

[97] Phan, K., Rao, P. J., Ball, J. R. & Mobbs, R. J. Interspinous process spacers versus traditional decompression for lumbar spinal stenosis: systematic review and meta-analysis. *J. Spine Surg.* **2**(1), 31–40. 10.21037/jss.2016.01.07 (2016). 27683693 10.21037/jss.2016.01.07 PMC5039840

[98] Sobottke, R. et al. Interspinous implants (X Stop®, Wallis®, Diam®) for the treatment of LSS: Is there a correlation between radiological parameters and clinical outcome?. *Eur. Spine J.* **18**(10), 1494–1503. 10.1007/s00586-009-1081-y (2009). 19562386 10.1007/s00586-009-1081-y PMC2899374

[99] Axle®—Xtant Medical. (n.d.). Xtant Medical. https://xtantmedical.com/product/axle/

[100] Yano, S. et al. A new ceramic interspinous process spacer for lumbar spinal canal stenosis. *Oper. Neurosurg.* **63**(1), 108–114. 10.1227/01.neu.0000310693.86660.d3 (2008). 10.1227/01.neu.0000335024.98863.19 18728587

[101] Rathbone, J. et al. A systematic review of anterior lumbar interbody fusion (ALIF) versus posterior lumbar interbody fusion (PLIF), transforaminal lumbar interbody fusion (TLIF), posterolateral lumbar fusion (PLF). *Eur. Spine J.* **32**(6), 1911–1926. 10.1007/s00586-023-07567-x (2023). 37071155 10.1007/s00586-023-07567-x

[102] Chatham, L. S., Patel, V. V., Yakacki, C. M. & Carpenter, R. D. Interbody spacer material properties and design conformity for reducing subsidence during lumbar interbody fusion. *J. Biomech. Eng.* **10**(1115/1), 4036312 (2017). 10.1115/1.4036312 PMC5446564 28334320

[103] Ito, M. et al. Evaluation of hydroxyapatite ceramic vertebral spacers with different porosities and their binding capability to the vertebral body: An experimental study in sheep. *J. Neurosurg. Spine* **6**(5), 431–437. 10.3171/spi.2007.6.5.431 (2007). 17542509 10.3171/spi.2007.6.5.431

[104] Rao, P. J., Shreshtha, N., Petersingham, G., Berg, A. & Seex, K. L2/3, L3/4 and L4/5 oblique lumbar interbody fusion/anterior to psoas: Anatomical and technical considerations. *Semin. Spine Surg.*10.1016/j.semss.2025.10116 (2025).

[105] Mondal, S. et al. Finite element analysis of vertebroplasty in the osteoporotic T11–L1 vertebral body: Effects of bone cement formulation. *J. Biomed. Mater. Res. Part B Appl. Biomater.*10.1002/jbm.b.35359 (2024). 10.1002/jbm.b.35359 38247244

[106] Long, Z., Zhou, J., Xiong, L., Chen, G. & Wen, J. Finite element study on three osteotomy methods for treating thoracolumbar osteoporotic fracture vertebral collapse complicated with neurological dysfunction. *Medicine* **103**(7), e36987. 10.1097/md.0000000000036987 (2024). 38363921 10.1097/MD.0000000000036987 PMC10869100

