# Supplementary material for: Computational analysis of L4–L5 interspinous process devices and interbody fusion spacers using ceramic and polymeric materials via finite element modeling and artificial intelligence
Source: Sci Rep. 2025 Oct 16;15:36142. doi: 10.1038/s41598-025-20870-5 (PMC12533109; doi:10.1038/s41598-025-20870-5)
Supplement: Supplementary file 1 — Supplementary Information. [file 41598_2025_20870_MOESM1_ESM.pdf]

Supplementary Information

Computational Analysis of L4–L5 Interspinous Process Devices and Interbody Fusion Spacers Using Ceramic and Polymeric Materials via Finite Element Modeling and Artificial Intelligence

Yomna H. Shash, Rana Hossam Elden

Supplementary Figures

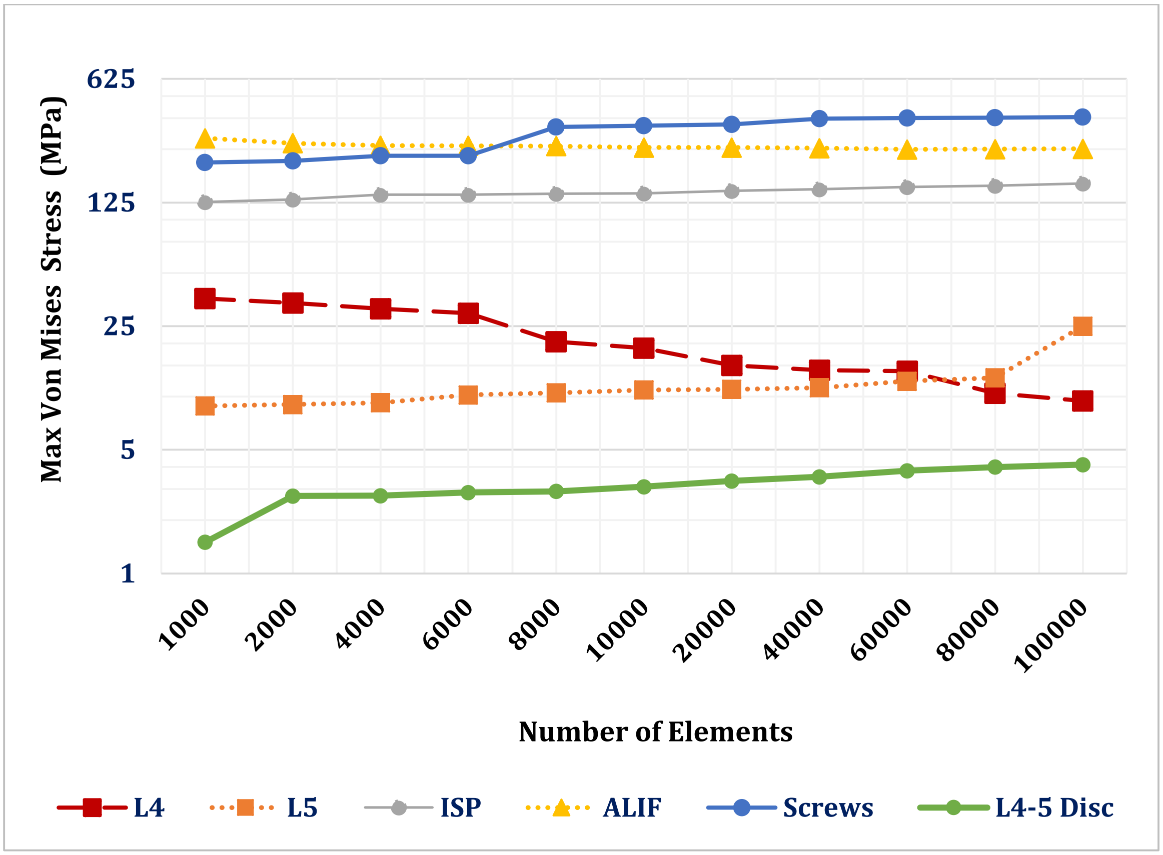

Figure S1: Convergence Test: The relation between number of elements and the maximum von Mises stresses on L4, L5, titanium ISP device, titanium ALIF cage, fixation screws and L4-5 disc, under flexion

Supplementary Tables

Table S1 : Sensitivity Analysis

Parameter Varied	Range	Change in max von Mises stress (%)	Change in L4-5 ROM (%)
• **Moment Magnitude**	± 10%	± 6.1%	± 6.9%
• **Friction coefficient of fact joints**	0.1-0.3	+5.1%	-3.6%
• **L4-L5 Disc Stiffness**	± 20%	± 4.5%	± 4.3%
• **Constraint settings**	full vs. partial fixations of L5	+ 2.5%	+ 1.8%

Table S2: Performance Metrics of Regression Models on ALIF Cages Dataset

Regression Model	MAE (MPa)	MSE $(MPa)^2$	RMSE (MPa)	R2	Running Time
**Fully Connected Network (MLP)**	44.4485	6699.4374	81.8501	0.9987	31.5852
**Support Vector Regression (SVR)**	313.606	329558.026	574.0714	0.9365	0.7336
**Random Forest Regression (RFR)**	391.6353	450968.892	671.5422	0.9132	8.6062
**XGBoost Regression (XGBR)**	230.7222	177508.415	421.3175	0.9658	7.7694
**LightGBM Regression (LGBMR)**	394.5441	456099.315	675.3513	0.9122	6.9289
**CatBoost Regression(CBR)**	392.637	454531.583	674.1896	0.9125	6.8608
**Bayesian Ridge Regression (BRR)**	765.8487	1851348.31	1360.643	0.6435	0.7448
**Polynomial Regression (PR), Degree = 2**	205.0984	165291.111	406.5601	0.9682	0.989
**Polynomial Regression(PR), Degree = 3**	205.0984	165291.111	406.5601	0.9682	0.5806
**Polynomial Regression(PR), Degree = 4**	205.0984	165291.111	406.5601	0.9682	0.6657
**K-Nearest Neighbors Regression(KNNR), K = 3**	450.8791	624975.4	790.5539	0.8797	0.5402
**K-Nearest Neighbors Regression(KNNR), K = 5**	493.1738	764107.129	874.1322	0.8529	1.233
**K-Nearest Neighbors Regression(KNNR), K = 7**	512.5219	791321.092	889.5623	0.8476	0.5152
**Gaussian Process Regression (GPR)**	0.1571	0.0403	0.2008	0.9993	1.7097
**Partial Least Squares Regression (PLSR)**	3.4537	14.556	3.8152	0.7307	0.2957
**Extreme Learning Machine (ELM)**	381.0165	477614.962	691.0969	0.908	0.3033
**Theil-Sen Estimator Regression(TSR)**	454.4222	658970.722	811.7701	0.8731	0.4592
**RANSAC Regression**	4.9149	35.4197	5.9514	0.3448	0.3698

Table S3: Performance Metrics of Regression Models on ISP Devices Dataset

Regression Model	MAE (MPa)	MSE $(MPa)^2$	RMSE (MPa)	R2	Running Time
**Fully Connected Network (MLP)**	54.7356	10033.28070	100.1663	0.9957	11.2692
**Support Vector Regression (SVR)**	194.3468	150707.0749	388.2101	0.9358	0.6478
**Random Forest Regression (RFR)**	227.5827	174703.1259	417.975	0.9255	5.8753
**XGBoost Regression (XGBR)**	127.9335	64338.7919	253.6509	0.9726	5.7730
**LightGBM Regression (LGBMR)**	224.7033	173817.0043	416.9137	0.9259	5.4416
**CatBoost Regression(CBR)**	227.6059	176160.8924	419.7153	0.9249	6.1870
**Bayesian Ridge Regression (BRR)**	375.8075	622197.5755	788.795	0.7348	0.5266
**Polynomial Regression (PR), Degree = 2**	134.4422	117224.346	342.3804	0.9500	0.5457
**Polynomial Regression(PR), Degree = 3**	134.4422	117224.346	342.3804	0.9500	0.3311
**Polynomial Regression(PR), Degree = 4**	134.4422	117224.346	342.3804	0.9500	0.3199
**K-Nearest Neighbors Regression(KNNR), K = 3**	284.031	254024.1525	504.0081	0.8917	1.7962
**K-Nearest Neighbors Regression(KNNR), K = 5**	336.6715	430530.8823	656.1485	0.8165	0.7354
**K-Nearest Neighbors Regression(KNNR), K = 7**	347.8615	469395.4575	685.1244	0.7999	0.5258
**Gaussian Process Regression (GPR)**	51.7717	10744.7521	103.6569	0.9954	6.9470
**Partial Least Squares Regression (PLSR)**	343.0574	343.0574	666.3857	0.8107	0.2081
**Extreme Learning Machine (ELM)**	327.498	438664.3128	662.3174	0.813	0.2470
**Theil-Sen Estimator Regression(TSR)**	280.9232	362504.9983	602.0839	0.8455	0.3127
**RANSAC Regression**	4.0498	18.0219	4.2452	0.8779	0.9890
